# Robust Melting Depression in Cu–Ag Despite Endothermic Mixing

**DOI:** 10.1021/acs.jpclett.6c02759

**Published:** 2026-10-14

**Authors:** Christopher M. Andolina, Wissam A. Saidi

**Affiliations:** Department of Mechanical Engineering and Materials Science, 6614 University of Pittsburgh, Pittsburgh, Pennsylvania 15261, United States

## Abstract

Cu–Ag exhibits a melting depression despite endothermic mixing and limited solid solubility. We combine density functional theory (DFT), machine-learning interatomic potentials, and large-scale two-phase coexistence simulations to resolve its composition-dependent melting, energetics, density, and transport. The lowest sampled fixed-composition coexistence temperature is 970 ± 3 K at 45 at. % Cu, while the maximum end point-referenced depression is 328.9 ± 2.5 K at 55 at. % Cu. The depression persists across ordered solids and prescribed solid–liquid composition partitions, with partitioning-induced shifts remaining modest relative to the depression. Intermediate composition solids incur a larger energetic penalty than the endothermically mixed liquid, providing an energetic basis for the melting depression without requiring favorable liquid mixing. PBE and PBEsol alter absolute melting temperatures, densities, and diffusivities but yield nearly identical maximum end point-referenced depressions. The depression is therefore robust to chemical ordering and functional choice, while its equilibrium depth depends on explicit solid–liquid compositional partitioning.

## Graphical abstract

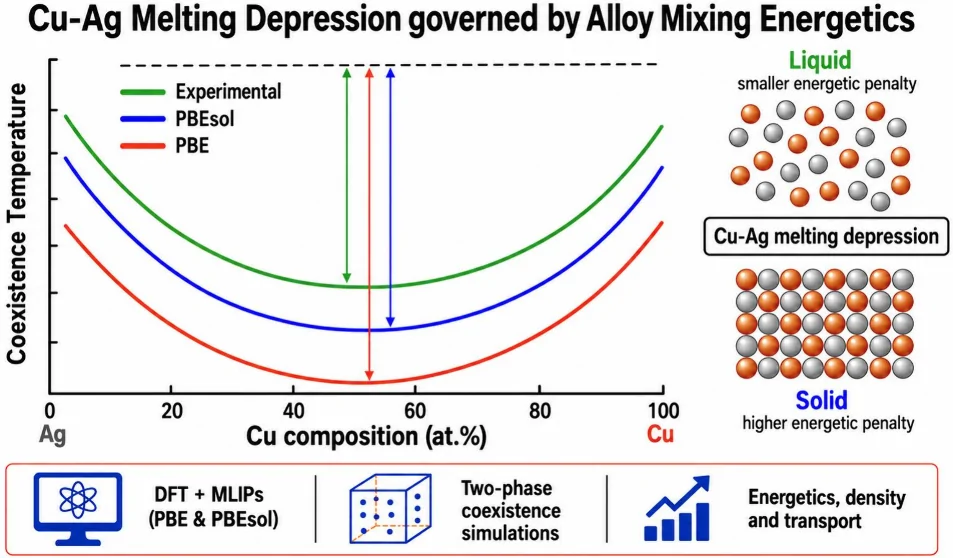

Eutectic melting stabilizes multicomponent liquids below the melting temperatures of their constituents and is important in alloy processing,^1^ thermal-energy storage,^2^ liquid-metal catalysis,^3^ and vapor–liquid–solid nanomaterial growth.^4^ Yet similar melting depressions need not have the same microscopic origin.^5^ In compound-forming alloys, favorable interactions between unlike atoms or competition among multiple crystalline compounds may shape the liquidus.^6^ In simple eutectics with restricted solid solubility, the relevant competition is instead between a disordered liquid and terminal solids that poorly accommodate unlike atoms. Stronger repulsion can destabilize even the liquid, as in Cu–Pb, which exhibits liquid immiscibility and monotectic behavior.^7^ A chemically simple system that remains homogeneous as a liquid is therefore valuable for isolating how a pronounced melting depression can persist despite unfavorable mixing.^8^

Eutectic melting is governed by free-energy competition, not by energetic stabilization of the mixed liquid alone. A liquidus minimum appears when the liquid free energy falls below the envelope defined by the solids with which it can coexist.^8^ Configurational entropy and structural freedom can favor the disordered liquid. In contrast, chemical mismatches, elastic strain, and restricted substitutional solubility can penalize intermediate-composition crystals. More generally, recent machine learning interatomic potential (MLIP)-based free-energy approaches are making anharmonic contributions to finite-temperature solid stability accessible at first-principles accuracy, emphasizing that static energetic trends alone are insufficient to establish phase boundaries.^9^ First-principles free-energy calculations illustrate the distinction: K–Na retains a homogeneous liquid and develops a deep eutectic even though mixing raises its internal energy, because the entropic contribution dominates; the larger energetic penalty in Li–Na instead produces a nonconvex liquid free energy and liquid–liquid phase separation.^5^ End-point melting temperatures, cohesive strength, and liquid mixing enthalpy therefore cannot individually determine the depth or even the topology of a eutectic phase diagram.

Cu–Ag provides an unusually clean metallic test of this competition. Both Cu and Ag metals form fcc crystals, no stable intermediate compound occurs in the assessed equilibrium phase diagram, and the eutectic liquid coexists with Ag-rich and Cu-rich terminal solid solutions of restricted mutual solubility. Historical Cu–Ag liquidus measurements span the composition range but exhibit appreciable scatter, particularly on the Ag-rich side. Roberts-Austen reported measurements of Cu–Ag alloy melting in 1875,^10^ followed by liquidus measurements using pyrometry,^11^ zone-refining/distribution-coefficient measurements,^12^ and electromotive force (emf) measurements.^13^ Subramanian and Perepezko subsequently assessed these data and selected a eutectic at 1052.2 K and 39.9 at. % Cu, with pure-Ag and pure-Cu melting points of 1234.9 and 1357.8 K, respectively.^14^ At the eutectic temperature, the assessed Ag-rich terminal fcc solid contains approximately 14.1 at. % Cu, while the Cu-rich terminal fcc solid contains approximately 4.9 at. % Ag (95.1 at. % Cu), highlighting the substantial equilibrium partitioning between the liquid and the two terminal solid solutions. Approximately at 40 at. % Cu, these two experimental references differ by ∼92 K (Figure 1). Nevertheless, both establish a pronounced intermediate-composition melting depression. At the same time, calorimetry shows that liquid Cu–Ag mixing is endothermic.^15^ Cu–Ag thus raises the question of how a deep, composition-dependent melting depression emerges without either a stable intermediate compound or a favorable net mixing enthalpy.

**Figure 1:**
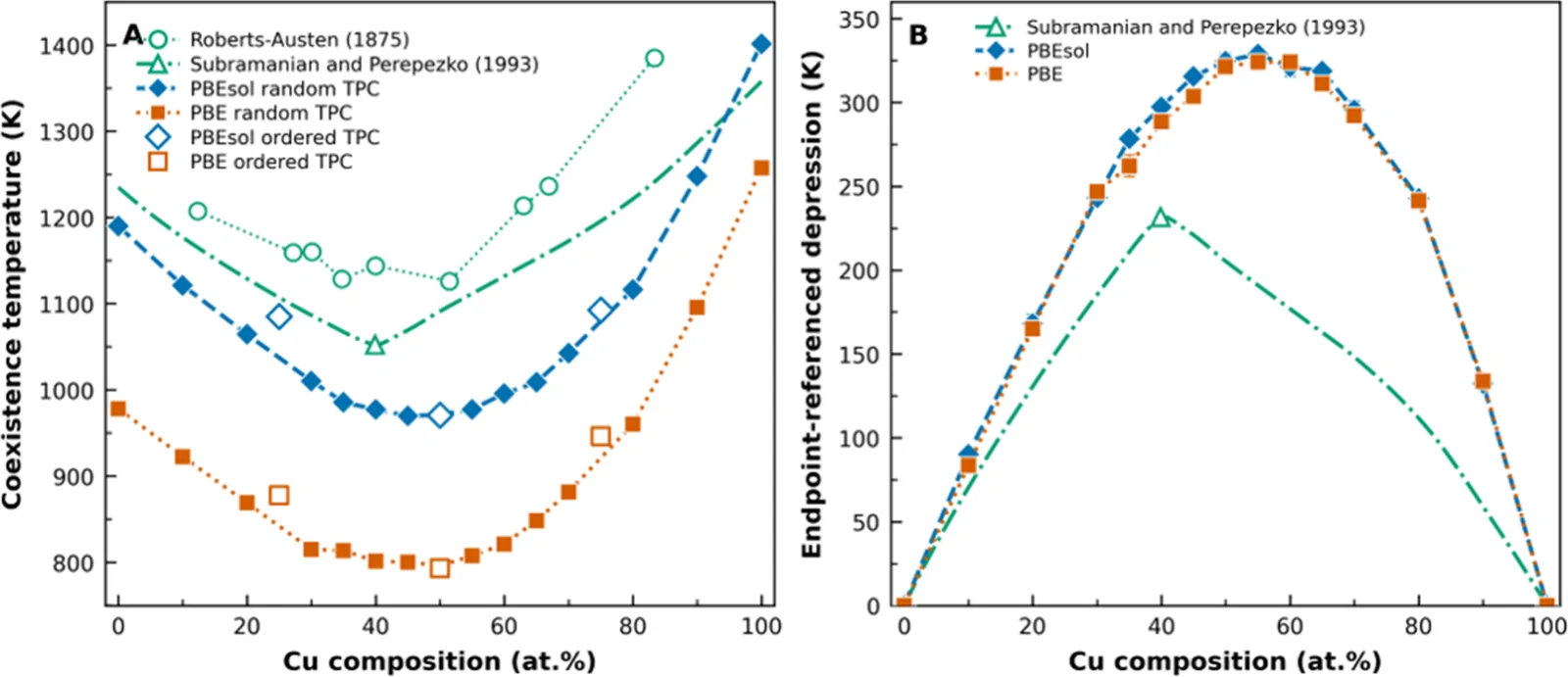
Composition-dependent melting of Cu–Ag. (A) TPC coexistence temperatures for random-alloy solids calculated with PBEsol and PBE, compared with the historical Roberts-Austen measurements and the assessed liquidus of Subramanian and Perepezko; the assessed eutectic is indicated explicitly. Ordered configurations are shown at 25, 50, and 75 at. % Cu. (B) End point-referenced depression, evaluated for each dataset relative to the linear interpolation between its corresponding pure-element end points. Error bars represent the standard error of the mean from replicate simulations and may be smaller than the symbols.

A recent PBE-based moment tensor potential (MTP) study by Nitol et al. provides an important complementary description of Cu–Ag phase behavior.^16^ Their work emphasized broad potential transferability—including defect, surface, stacking-fault, phonon, ordered-structure, and melting properties—and reconstructed the equilibrium binary phase diagram using semi-Grand-Canonical sampling that explicitly permits Cu–Ag chemical exchange. The resulting eutectic was predicted at 1000.84 K and approximately 43 at. % Cu.^16^ Thus, the semi-Grand-Canonical MLIP calculation provides an equilibrium computational analog of the assessed phase diagram, reproducing an intermediate-composition eutectic bounded by Ag-rich and Cu-rich terminal fcc solid solutions, while shifting the absolute eutectic temperature downward relative to the assessment. By permitting equilibrium solid-liquid partitioning, this approach yields both solidus and liquidus boundaries. The present work addresses a different question: rather than reconstructing the equilibrium phase diagram with a single PBE-based potential, we use compositionally constrained two-phase coexistence (TPC), controlled ordering and partitioning perturbations, and parallel PBE/PBEsol models to identify which energetic, structural, and functional-dependent features generate the melting depression and which aspects remain robust when the absolute thermodynamic scale changes. The distinction between these approaches is thermodynamic as well as methodological. The present equal-composition TPC calculations determine constrained coexistence temperatures in which the solid and liquid retain the same nominal composition. In contrast, determining equilibrium solidus and liquidus boundaries requires equality of the chemical potentials of each component across the coexisting phases.

Machine-learning interatomic potentials are increasingly enabling composition-resolved simulations of alloy thermodynamics and melting at length and time scales inaccessible to direct first-principles molecular dynamics.^16–21^ Here, we develop first-principles-,^22–24^ PBE-,^25^ and PBEsol^26^-based MLIPs,^27^ and apply them in TPC molecular dynamics (MD) simulations^17,19,21,28^ across the Cu–Ag composition range. Model validation, literature benchmarking,^18,20,29,30^ and finite-temperature ensemble-deviation tests are summarized in Figure S1 and Tables S1–S3. TPC directly represents the crystalline and liquid regions and identifies the temperature at which neither phase systematically grows at the expense of the other. Such system-specific validation remains important even as transferable and foundational MLIP architectures become increasingly capable, because fine-tuning can substantially improve quantitative accuracy for targeted materials and properties.^31^ Guided by previous convergence studies, we employ large cells and extended trajectories to reduce finite-size effects, interfacial constraints, and thermal noise; cells containing fewer than approximately 1000 atoms can incur melting-temperature errors of up to 100 K.^19,28^ The primary calculations assign the same nominal composition to the solid and liquid. Ordered crystals test sensitivity to chemical ordering, while deliberately mismatched solid–liquid compositions assess sensitivity to compositional partitioning. PBEsol provides the primary description, with PBE being used to assess whether the conclusions hold despite functional sensitivity across the absolute melting, structural, and transport scales.

PBEsol predicts melting temperatures of 1190.2 K for pure Ag and 1401.5 K for pure Cu, compared with the assessed experimental values of 1234.9 and 1357.8 K, respectively (Figure 1A). Thus, both calculated end points agree with experiment within approximately 45 K, although Ag is underestimated and Cu is overestimated. These values are also consistent with previous MLIP predictions based on PBE- and PBEsol-derived training datasets using both MTPs^16,21^ and deep neural-network potentials (DNPs).^19^ In particular, the present PBE end point temperatures of 978.2 K for Ag and 1257.3 K for Cu agree closely with the independently trained PBE MTP results of Wisesa et al. (978 and 1243 K, respectively), differing by less than 1 K for Ag and 14 K for Cu.^21^ The PBEsol agreement is similarly good: the present values differ by only approximately 25 K for Ag and 30 K for Cu from the corresponding PBEsol MTP predictions of 1215 and 1432 K.^21^

The spread among independently developed PBE MLIPs is also informative. Nitol et al. obtained MTP melting temperatures of approximately 1125 K for Ag and 1332 K for Cu, substantially higher than the present PBE DNP values of 978.2 and 1257.3 K, respectively, while their equilibrium phase-diagram calculation yielded a eutectic at 1000.84 K near 43 at. % Cu.^16^ Their MTP used a different training and validation strategy, and they obtained the binary phase diagram using chemical-potential-controlled sampling rather than fixed-composition NPH (constant particle number, pressure, and enthalpy) coexistence. These comparisons separate two effects that should not be conflated: MLIP construction can shift the absolute PBE melting scale, whereas allowing equilibrium chemical partitioning changes the thermodynamic quantity being calculated. The Nitol et al. eutectic is an equilibrium phase-boundary prediction, whereas the present intermediate-composition TPC values are deliberately constrained coexistence benchmarks.^16^ Nevertheless, both approaches predict a broad Cu–Ag melting depression, supporting the robustness of the underlying behavior.

To determine how alloying modifies this end point behavior, we constructed random-alloy TPC cells from pure Ag to pure Cu at 10 at. % composition intervals, with additional sampling at 5 at. % intervals near the minimum. At each intermediate composition, the crystalline region was a substitutionally random fcc alloy and the liquid region had the same nominal composition. The lowest sampled PBEsol coexistence temperature is 970 ± 3 K at 45 at. % Cu (Figure 1A). Relative to the linear interpolation between the calculated end points, the largest sampled depression is approximately 329 K at 55 at. % Cu (Figure 1B).

Independent initial-velocity trajectories were propagated between 90 and 100 ps under NPH conditions at standard pressure. Spatially resolved structural-order profiles identified persistent coexistence and excluded trajectories that lost the interface or recrystallized completely (Table S4 and Figure S2). Composition-level standard errors are 0.4–8.1 K, much smaller than the approximately 330 K maximum end-point-referenced depression. Additional cohesive-energy/melting diagnostics and the end point-referenced depression summary are provided in Figure S3 and Table S5.

Because the primary TPC solids are substitutionally disordered, we evaluated whether random chemical occupancy artificially destabilizes the solid and produces the melting depression. L1_2_ CuAg_3_ and Cu_3_Ag structures, and an equiatomic fcc-based ordering, shift the coexistence temperatures at selected compositions but, importantly, do not eliminate the broad melting minimum (Figure 1). These ordered configurations are hypothetical structural sensitivity tests rather than proposed equilibrium phases. Consistent with this interpretation, the assessed experimental Cu–Ag phase diagram contains no stable intermetallic compound; only Ag-rich and Cu-rich terminal fcc solid solutions coexist with the eutectic liquid.

The equal-composition construction isolates how disorder changes the relative stability of a mixed liquid and mixed crystal at the same nominal composition. Still, the resulting constrained coexistence temperature is not an equilibrium liquidus point. At the assessed eutectic, the equilibrium terminal solids contain approximately 14.1 and 95.1 at. % Cu on the Ag-rich and Cu-rich sides, respectively. The equal-composition TPC construction therefore suppresses the substantial compositional partitioning present in the equilibrium phase diagram. Constraining the two phases to the same nominal composition prevents equilibrium partitioning and can therefore shift the calculated coexistence temperatures relative to the experimental liquidus.

We therefore assessed sensitivity to the imposed partition using nine PBEsol TPC cells at overall compositions of 40, 50, and 60 at. % Cu (Figure 2). Each overall composition was represented by three mismatched solid–liquid pairs while conserving the total Cu fraction. For example, the cells containing 50 at. % Cu overall were initialized with solid/liquid compositions of 40/60, 30/70, and 20/80 at. % Cu, respectively. After 300 ps of NPH dynamics, the Cu compositions changed by no more than 0.7% in the solid and 2.0% in the liquid (see Figure 2A, as well as Figure S4 and Table S6). Correspondingly, 97%–103% of the initially imposed solid–liquid composition difference is retained across the nine calculations, demonstrating that direct MD-TPC retains substantial compositional memory on the simulated time scale. Spatially resolved Cu concentration profiles provide a complementary check on this compositional retention (Figure S10). The three 50 at. % overall-composition systems were initialized at solid/liquid Cu compositions of 20/80, 30/70, and 40/60 at. %. Their late-time bulk compositions are 19.4/80.1, 30.5/70.9, and 40.6/60.0 at. % Cu, respectively, with the composition changes localized primarily across the diffuse solid–liquid interfacial regions.

**Figure 2:**
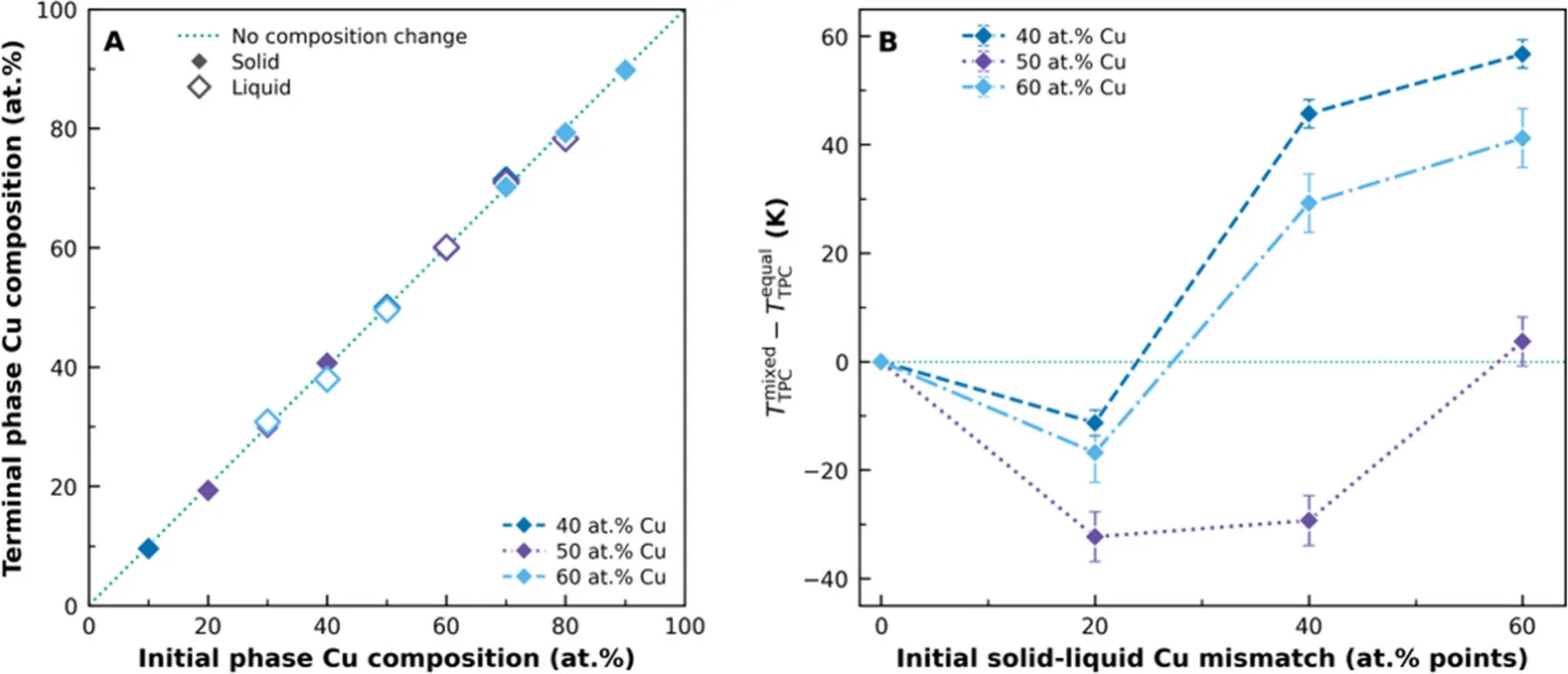
Sensitivity of TPC results to the imposed solid–liquid composition partition. PBEsol simulations were performed at overall compositions of 40, 50, and 60 at. % Cu. (A) Terminal versus initial Cu compositions of the solid and liquid regions; the diagonal line indicates unchanged composition. (B) Coexistence-temperature shift relative to the corresponding equal-composition calculation versus the initial solid–liquid mismatch. Error bars combine the standard errors of the mismatched- and equal-composition calculations.

Because the imposed mismatches remain essentially unchanged, each calculation probes coexistence for a specified solid–liquid composition pair. Relative to the corresponding equal-composition calculations, these prescribed partitions shift the inferred coexistence temperature by −32 to +57 K (Figure 2B). The shift depends on both the overall composition and the selected solid–liquid pair, rather than varying monotonically with the mismatch magnitude. Thus, partitioning modifies but does not eliminate the pronounced melting depression. Nevertheless, these coexistence temperatures are sensitivity measurements rather than equilibrium liquidus points because potential-energy differences alone cannot determine the equilibrium partition. Semi-Grand-Canonical sampling provides a complementary route by allowing chemical exchange and directly constructing equilibrium phase boundaries.^16^

The persistence of the minimum across random and ordered solids rules out a single-occupancy origin. It motivates a direct comparison of the energetic penalties between the competing phases. For PBEsol, the random substitutional fcc solids bow unfavorably relative to the cohesive-energy tie line connecting pure Ag and Cu (Figure 3A). Because cohesive energy is plotted as a positive binding magnitude, the downward bowing reflects a solid-state penalty of approximately 100 meV/atom near equiatomic composition. The ordered configurations remain close to the random-alloy trend and do not create a compound-like energetic minimum, showing that chemical ordering neither stabilizes an intermediate compound nor removes the melting depression.

**Figure 3:**
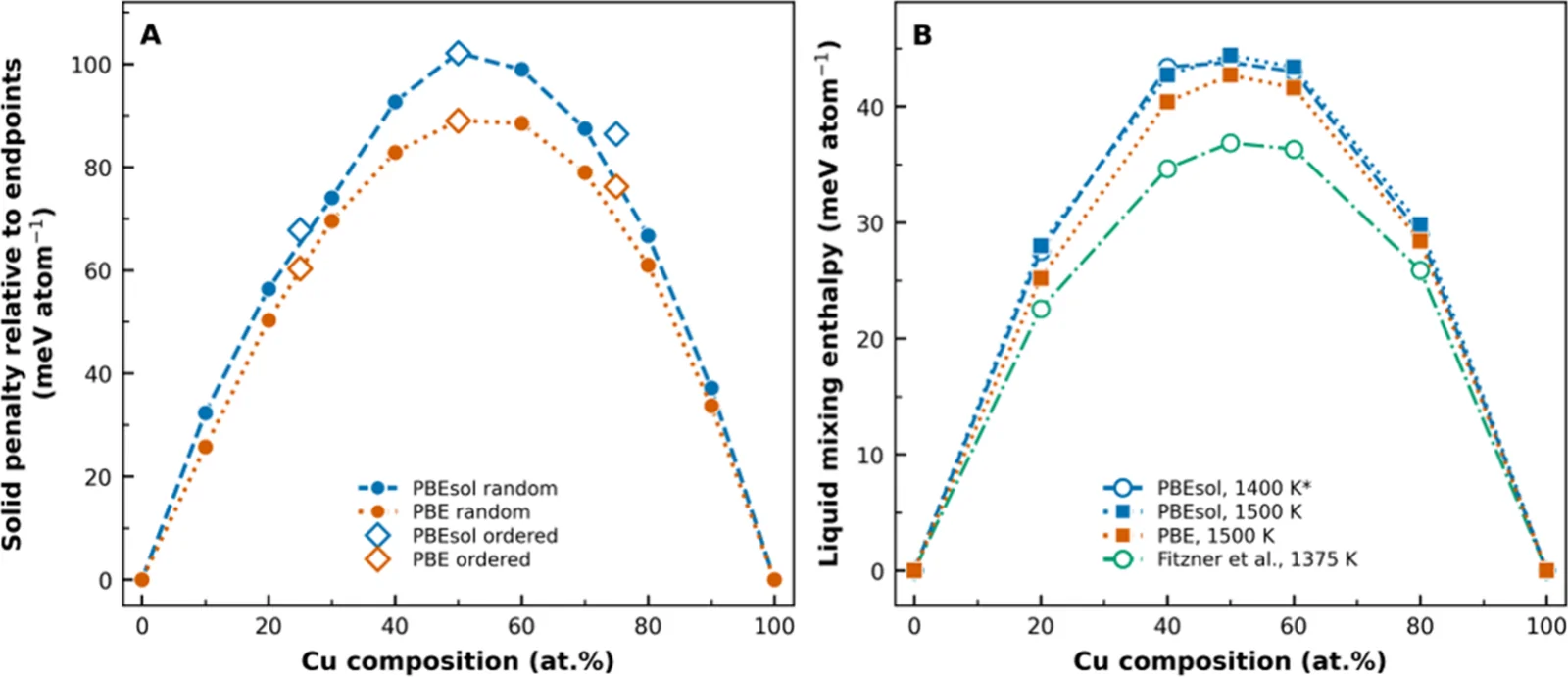
Energetic competition between Cu–Ag solids and liquids. (A) Penalty of random substitutional fcc solids relative to the pure-end point cohesive-energy tie line; open diamonds denote ordered configurations. (B) Liquid mixing enthalpy at 1400 and 1500 K compared with the experimental representation of Fitzner et al. at 1375 K. Error bars represent standard errors and may be smaller than the symbols.

Liquid mixing enthalpies provide the complementary comparison (Figure 3B). At 1500 K, the PBEsol mixing enthalpy peaks at 44.4 meV/atom near the equiatomic composition; the 1400 K curve reaches a similar value of 43.8 meV/atom using the estimated metastable liquid-Cu end point. The experimental representation from Fitzner et al. gives 36.8 meV/atom near equiatomic composition at 1375 K.^15^ The positive values confirm endothermic liquid mixing and exclude favorable net liquid mixing as the origin of the depression. Although the solid and liquid energetic benchmarks are evaluated at different temperatures and are not directly comparable free energies, both show unfavorable intermediate-composition energetics, with the solid penalty larger in their respective benchmark.

Figure 3 therefore resolves the energetic aspect of the apparent paradox: neither mixed state is energetically stabilized, yet the solid incurs the stronger penalty. A eutectic minimum requires the liquid free energy to fall below the relevant solid free-energy envelope; it does not require favorable liquid mixing relative to the pure liquids. K–Na provides a useful precedent: first-principles calculations showed that entropy overcomes its positive internal-energy penalty and preserves a homogeneous liquid, whereas the larger penalty in Li–Na produces liquid–liquid separation.^5^ Cu–Ag similarly retains a mixed liquid despite endothermic mixing, while intermediate-composition solids incur a larger energetic penalty. Equilibrium partitioning into Ag-rich and Cu-rich terminal solids further modifies the relevant solid free-energy envelope.^32^

The energetic interpretation would be less compelling if it depended on a single exchange–correlation approximation. We therefore repeated the composition-dependent TPC calculations with PBE. PBE lowers the Ag and Cu coexistence end points to 978.2 and 1257.3 K, respectively, compared with 1190.2 and 1401.5 K for PBEsol (Figure 1A). Nevertheless, the maximum sampled end point-referenced depression is 324.2 ± 2.2 K for PBE, closely comparable to 328.9 ± 2.5 K for PBEsol (Figure 1B). Relative depression therefore persists despite the strong functional sensitivity of the absolute melting scale. More-detailed temperature-dependent density and enthalpy trends, along with selected numerical values, are provided in Figure S7 and Table S9.

That robustness is striking because PBE and PBEsol describe different zero-temperature bonding and compression scales. Across Ag, Cu_25_Ag_75_, Cu_50_Ag_50_, Cu_75_Ag_25_, and Cu, PBEsol systematically shifts the equation of state (EOS) toward smaller equilibrium volumes and larger bulk moduli (see Figures S5 and S6, as well as Tables S7 and S8). For example, replacing PBE with PBEsol decreases the DFT equilibrium volume from 17.83 Å^3^ atom^–1^ to 16.62 Å^3^ atom^–1^ for Ag, from 15.11 Å^3^ atom^–1^ to 14.21 Å^3^ atom^–1^ for Cu_50_Ag_50_, and from 12.00 to 11.35 Å^3^ atom^–1^ for Cu, while increasing the corresponding bulk moduli from 88.2 to 118.4, 109.2 to 135.7, and 137.7 to 164.5 GPa, respectively. The DNP EOS curves closely reproduce these functional-dependent DFT trends, and the pointwise DNP–DFT deviations remain minor compared with the separation between the two functionals (see Figures S5 and S6, as well as Tables S7 and S8). Thus, the functional dependence observed in the melting calculations reflects a genuine difference in the parent electronic-structure description rather than a surrogate-potential artifact.

Cohesive-energy diagnostics provide a complementary perspective on why this substantial change in absolute bonding strength does not uniquely determine the alloy melting behavior. Stronger cohesion generally accompanies the higher PBEsol melting scale, and the elemental end points therefore retain the expected correlation between cohesive strength and melting temperature (*T*_m_). However, this correlation breaks down as a predictor of the composition-dependent depression. Plotting the calculated coexistence temperatures against the 0 K cohesive energies or using an end-point-calibrated cohesive-energy estimate of *T*_m_ reproduces the broad end point ordering but does not generate the deep intermediate-composition minimum observed directly in TPC (Figure S3). The failure is consistent with the end point-referenced depressions of 324.2 ± 2.2 K for PBE and 328.9 ± 2.5 K for PBEsol (Table S5): cohesive energy establishes an important absolute energetic scale, but it contains neither the finite-temperature entropy nor the distinct solid–liquid free-energy response required to determine the liquidus. In this sense, the EOS and cohesive-energy results reinforce the central picture of Figure 3: absolute bonding strength strongly shifts the melting-temperature scale, whereas the depression itself emerges from the relative thermodynamics of the competing solid and liquid states.

To assess whether the unfavorable solid-state energetics persist at finite temperature, we performed additional constant particle number, pressure, and temperature (NPT) molecular-dynamics calculations for the ordered Cu–Ag configurations at 700 and 800 K. The mixing enthalpies remain positive at all intermediate compositions for both functionals (see Figure S11 and Table S12). At 50 at. % Cu, PBE predicts 135.6 ± 0.8 and 134.0 ± 0.7 meV atom^–1^ at 700 and 800 K, respectively, compared with 134.1 ± 0.9 and 133.8 ± 0.8 meV atom^–1^ for PBEsol. All final configurations retained their initial crystalline ordering upon visual inspection. These results establish that the substantial energetic penalty associated with the equiatomic ordered solid persists at finite temperature and exhibits little functional dependence. The zero-point-energy mixing correction at 50 at. % Cu is small (−3.11 meV atom^–1^ for PBE and −3.66 meV atom^–1^ for PBEsol), leaving the corrected mixing enthalpy strongly positive.

In the liquid transport simulations, PBE predicts a less dense, more mobile liquid, whereas PBEsol predicts a denser, less mobile liquid. Shear viscosity provides an additional transport benchmark (Figure 4D). At 1375 K, PBEsol predicts higher viscosities than PBE across all compositions for which both functional-specific results are available. At 1475 K, the functional separation is smaller over most of the composition range, although a larger difference remains at the pure-Cu end point. Comparison with the available experimental data is complicated by the substantial experimental and Green–Kubo uncertainties,^33,34^ limiting quantitative discrimination between the functionals. The PBEsol pure-Cu viscosity at 1375 K is unavailable and is omitted from the corresponding comparison. Differences in relative superheating preclude attributing the transport behavior solely to liquid density. Numerical results and Green–Kubo convergence diagnostics are provided in the Supporting Information (see Figure S9 and Table S11).^35^

**Figure 4:**
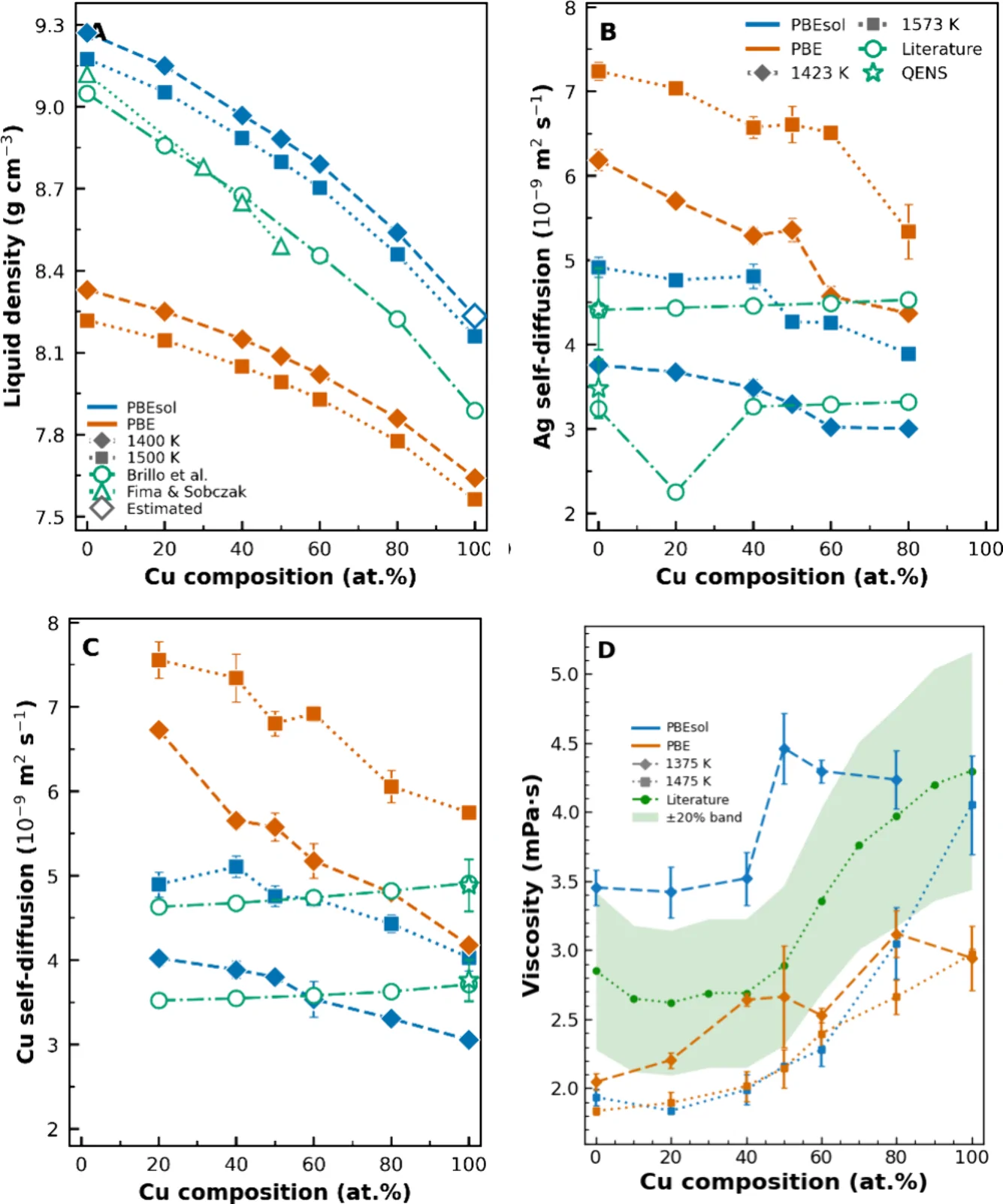
(A) Liquid density at 1400 and 1500 K, compared with literature values measured near 1373 K;^39,40^ the open PBEsol Cu end point at 1400 K is estimated. (B,C) Self-diffusion coefficients of (B) Ag and (C) Cu at 1423 and 1573 K compared with literature^36^ and quasielastic neutron scattering (QENS) values.^37^ (D) Shear viscosity at 1375 and 1475 K from Green–Kubo analysis compared with available experimental values from Brillo et al. at 1375 K; the shaded band indicates ±20% around the experimental trend, and the PBEsol 1375 K pure-Cu end point is omitted because it is unavailable.

Self-diffusion provides a dynamical counterpart (Figure 4B,C). Diffusivity increases with temperature and varies smoothly with composition for both species, without an anomaly near the melting minimum. Across 20–80 at. % Cu, PBEsol values are 31%–40% lower than PBE at 1423 K and 27%–35% lower at 1573 K. Available literature data and quasielastic neutron scattering (QENS) end point measurements provide reference values for both species.^36–38^ The QENS end points place liquid Cu between the functional predictions and show closer agreement with PBEsol for liquid Ag. Representative mean-square displacement (MSD) fits and the complete composition-dependent diffusion values are given in Figure S8 and Table S10.

Cu–Ag exhibits a pronounced intermediate-composition melting depression despite unfavorable solid-state binding and positive liquid mixing enthalpy. Random and ordered structural models show that the depression is neither an artifact of random chemical occupancy nor the signature of an ordered intermediate compound. Prescribed solid–liquid composition pairs shift the inferred coexistence temperatures, while direct molecular-dynamics TPC retains substantial compositional memory over the simulated time scale. The fixed-composition calculations therefore characterize the competition between mixed solid and liquid states, whereas the equilibrium liquidus additionally depends on solid–liquid compositional partitioning.

The solid and liquid energetics demonstrate that the melting depression does not require favorable liquid mixing. Changing the exchange–correlation functional shifts absolute coexistence temperatures, liquid densities, and diffusivities, but preserves the broad intermediate-composition depression and a nearly unchanged end point-referenced magnitude. Additional finite-temperature calculations confirm that ordered crystalline Cu–Ag remains strongly endothermic upon mixing. The combined results therefore distinguish robust qualitative behavior from quantitative sensitivity: chemical ordering and functional choice do not eliminate the depression, whereas solid–liquid compositional partitioning affects its quantitative depth and must be treated explicitly to recover the equilibrium liquidus.

## Supplementary Material



## Data Availability

The trained potentials, simulation input files, analysis scripts, and additional supporting data are publicly available in the Saidi Research Group GitHub repository “CuAg_alloy” at https://github.com/saidigroup/CuAg_alloy.

## Notes

Computational methods; neural-network-potential training, validation, adaptive-learning coverage, and literature benchmarking; equation-of-state fits and pointwise surrogate diagnostics; finite-temperature ordered-solid enthalpy and harmonic phonon/vibrational-mixing analyses; spatially screened fixed-composition coexistence trajectories and replicate data; cohesive-energy and melting diagnostics; complete density, enthalpy, diffusion, and viscosity datasets; representative MSD and Green–Kubo convergence analyses (PDF)
