# Supplementary material for: Robust Melting Depression in Cu–Ag Despite Endothermic Mixing
Source: J Phys Chem Lett. 2026 Oct 14;17(43):12483–90. doi: 10.1021/acs.jpclett.6c02759 (PMC13726461; doi:10.1021/acs.jpclett.6c02759)
Supplement: Supplementary file 1 [file jz6c02759_si_001.pdf]

Supporting Information:

Robust Melting Depression in Cu–Ag despite Endothermic Mixing

Christopher M. Andolina and Wissam A. Saidi*

Department of Mechanical Engineering and Materials Science, University of Pittsburgh, Pittsburgh, PA 15261, USA.

*Corresponding author: alsaidi@pitt.edu

TABLE OF CONTENTS

Computational Methods 2
DFT Reference Calculations 2
Neural-Network Potential Training and Validation 2
Network Architecture 2
Equation-of-State and Cohesive-Energy Analysis 3
Harmonic Phonon Calculations 3
Fixed-Composition Solid-Liquid Coexistence 4
Density and Enthalpy 5
Finite-Temperature Ordered-Solid Mixing Enthalpies 5
Self-Diffusion and Viscosity 6
Neural-Network Potential Validation 6
Fixed-Composition Melting and Cohesive-Energy Diagnostics 12
Composition-Mismatched Coexistence Calculations 17
Equation-of-State and Surrogate Validation 21
Thermophysical and Transport Diagnostics 28
Additional Spatial Composition Profiles 34
Finite-Temperature Solid Mixing and Harmonic Vibrational Thermodynamics 35
REFERENCES 40

Computational Methods

DFT Reference Calculations. Density-functional theory (DFT) calculations were performed with the Vienna ab initio Simulation Package (VASP 6.4.1)1, 2 using the projector-augmented-wave (PAW) method3. Exchange-correlation effects were evaluated with PBE4 and PBEsol5. The PAW datasets were PAW_PBE Cu 22Jun2005 and PAW_PBE Ag 02Apr2005. Calculations used a 400 eV plane-wave cutoff, KSPACING = 0.24, Methfessel-Paxton smearing with ISMEAR = 1 and SIGMA = 0.15 eV, and an electronic convergence threshold of 1×10^{-7} eV for static equation-of-state (EOS) calculations.

Neural-Network Potential Training and Validation. The Cu-Ag neural-network potentials were trained with DeePMD-kit 2.2.10 using functional-specific PBE and PBEsol DFT reference datasets.[6] The PBE model series reused previously reported Cu-Ag PBE training data [7] and the PBEsol series was generated with an analogous reference-data workflow. Structures were filtered for electronic convergence and force/energy outliers, grouped by composition and structure class, and split into training and validation sets. Adaptive learning used independently seeded three-model ensembles to identify configurations with elevated model-force deviations; selected frames were recomputed with the corresponding DFT functional and added to later training iterations. Final ensemble tests sampled 108-atom configurations at Cu compositions from 0 to 100 at.% in 10 at.% increments, and at 400, 800, 1200, and 1600 K. Each test used 10 ps of constant particle number, pressure, and temperature (NPT) equilibration at 1.01325 bar followed by a 40 ps constant particle number, volume, and temperature (NVT) trajectory, with model-deviation statistics recorded every 2 ps.

The validation metrics establish that the surrogate models reproduce their parent functionals with sufficient accuracy for the melting, thermodynamic, and transport comparisons. They should not be interpreted as proving transferability outside the sampled Cu-Ag domain. Figure S1 shows validation-error learning curves for the independently trained model ensembles, Table S2 places the errors in the context of selected validated alloy ML-IAPs, and Table S3 summarizes both dataset coverage and the final finite-temperature ensemble-deviation tests. The model-deviation values quantify disagreement among independently trained models and are uncertainty indicators rather than direct DFT errors. The purpose is to show that surrogate errors and ensemble disagreement are small relative to the functional-dependent property shifts, not to make model architecture the focus of the paper.

Network Architecture. The final PBE and PBEsol model sets used a DeepPot-SE descriptor with a 9 Å cutoff, embedding-network dimensions of 32, 64, and 100, and fitting-network dimensions of 512, 512, and 512. Models were trained for 3.2×10^{6} steps with the learning rate decayed from 1×10^{-3} to 5×10^{-8}. The principal production models were Cu_Ag-9-x/Cu_Ag-9-0.pb for PBE and Cu_Ag-10-x/Cu_Ag-10-1.pb for PBEsol. Model files ending in “c” are DeePMD-compressed deployable forms of the corresponding frozen models, not independently trained fits. Validation RMSEs are summarized in Table S1, and dataset-system coverage and finite-temperature ensemble-deviation tests are summarized in Table S3.

Equation-of-State and Cohesive-Energy Analysis. Relaxed Cu-Ag structures were isotropically scaled to volume factors of 0.94, 0.955, 0.97, 0.985, 1.00, 1.015, 1.03, 1.045, and 1.06. Static DFT calculations were performed at each fixed volume using NSW = 0 and ISIF = 2. We considered Ag, $Cu_{25}Ag_{75}$, $Cu_{50}Ag_{50}$, $Cu_{75}Ag_{25}$, and Cu. Corresponding surrogate calculations used the same imposed cell volumes and fixed-cell atomic minimization before energy extraction. Energies for each composition, functional, and method were shifted relative to the minimum of the corresponding series. EOS data were fitted with the standard third-order Birch-Murnaghan equation of state [8, 9]. The pressure derivative B0′ is dimensionless; equilibrium energies and volumes are reported per atom in Table S7, and pointwise data are provided in Table S8.

Ordered-structure metadata. The ordered/intermetallic-like sensitivity tests used 6912-atom FCC-based cells. The 25 at.% Cu structure was $L1_2$ $CuAg_3$ (1728 Cu and 5184 Ag atoms), and the 75 at.% Cu structure was $L1_2$ Cu_3Ag (5184 Cu and 1728 Ag atoms); the supplied coordinates and small cell tilts indicate relaxed/distorted cells. The equiatomic structure contained 3456 Cu and 3456 Ag atoms in a FCC occupancy generated on an ideal cubic starting lattice. These configurations were used as structural sensitivity tests and were not treated as stable Cu-Ag compounds.

Harmonic Phonon Calculations. Harmonic phonon calculations were performed using Phonopy (version 2.41.3) and the PBE- and PBEsol-trained deep neural-network potentials (DNPs). We performed calculations for pure Ag, $Cu_{25}Ag_{75}$, $Cu_{50}Ag_{50}$, $Cu_{75}Ag_{25}$, and pure Cu using relaxed 32-atom reference structures. Finite-displacement force constants were calculated using 4 × 4 × 4 supercells containing 2,048 atoms. Additional PBEsol 6 × 6 × 6 supercell calculations, containing 6,912 atoms, were used to assess supercell-size convergence. Symmetry-reduced atomic displacements of 0.01 Å were generated using Phonopy. Forces on the displaced configurations were evaluated using LAMMPS coupled to the corresponding functional-specific DeePMD potential. The resulting forces were used to construct the harmonic force constants. Phonon frequencies and vibrational thermodynamic properties were evaluated using a 30 × 30 × 30 reciprocal-space mesh. Frequencies below −0.10 THz were counted as imaginary modes for the stability diagnostic. Vibrational free energies, entropies, and constant-volume heat capacities were calculated from 0 to 1600 K in increments of 10 K. The vibrational mixing free energy per atom was defined as

$$\Delta F_{vib,mix}(x,T) = F_{vib}(Cu_xAg_{1-x},T) - xF_{vib}(Cu,T) - (1-x)F_{vib}(Ag,T) \quad (S1)$$

where x denotes the atomic fraction of Cu (equation S1). All terms were normalized per atom and evaluated at the same temperature using the same exchange–correlation functional. Vibrational mixing entropies were calculated analogously from the corresponding harmonic entropy values. These quantities describe the harmonic vibrational contribution to mixing within the crystalline state. They do not include configurational entropy or provide a complete solid–liquid free-energy difference.

Fixed-Composition Solid-Liquid Coexistence. Production molecular-dynamics (MD) calculations were performed with LAMMPS (2 Aug 2023 - Update 1) coupled to USER-DEEPMD. Training employed DeePMD-kit 2.2.106, 10, using LAMMPS11. The two-phase protocol, simulation lengths, and the reasonableness of comparable large-cell dimensions follow prior machine-learning interatomic potential (MLIP) two-phase coexistence (TPC) validation studies7, 12, 13 ; a separate methodological finite-size study was therefore not repeated for Cu-Ag. Two-phase coexistence simulations used 13,824-atom cells generated from 12 × 12 × 24 replications of the FCC conventional cell. The long cell direction was oriented along z, giving approximate cell dimensions of 50 × 50 × 100 Å with the solid-liquid interface normal parallel to z. Simulations were performed at standard pressure (1.01325 bar). After energy minimization, the crystal was equilibrated at the trial melting temperature for 5000 steps with a 4 fs time step (20 ps) in the NPT ensemble. Velocities in one half of the cell were then initialized at 1.40 * Tm, and that half was thermostatted at 1.35 * Tm for 6000 steps with a 2.5 fs time step (15 ps) to form the liquid slab. The liquid half was subsequently cooled at 1.03* Tm for 6000 steps with a 3 fs time step (18 ps). Final coexistence trajectories were targeted for 50,000 steps with a 2 fs time step (100 ps) in the NPH ensemble, with a pressure-damping time of 2 ps. Five retained PBEsol trajectories terminated after 45,000-

47,000 steps (90-94 ps) but were accepted because their terminal spatially resolved order profiles still exhibited two distinct solid-liquid interfaces. Replicates A, B, and C used the same composition and atomic occupancy but independent initial-velocity seeds; their scatter therefore reflects trajectory/velocity uncertainty rather than chemical-configuration uncertainty. We evaluated a 12-neighbor centrosymmetry parameter and used a CSP threshold of 2.5 for the structural-order analysis; we then averaged the resulting order metric in bins along z every 2500 steps (5 ps). We normalized each profile to its liquid- and solid-region plateaus, and identified retained coexistence by the presence of two spatially separated solid-liquid interfaces. The cell-averaged count below the centrosymmetry cutoff was not interpreted as a physical solid volume fraction. Runs that became spatially uniform and fully crystalline were excluded, whereas interface migration during latent-heat redistribution did not by itself invalidate a trajectory if both phases remained. Automated controller verdicts were not used in this physical screening. Reported coexistence temperatures are constrained fixed-composition benchmarks and do not impose equilibrium partitioning between the liquid and Ag-rich or Cu-rich solids.

Species-partitioning check. Terminal snapshots from retained random-alloy trajectories at 40, 50, and 60 at.% Cu were binned along z and compared with the corresponding normalized local-order profiles. Bins assigned to the bulk solid and liquid regions differed in Cu fraction by less than 1 at.% for every resolved trajectory, including the updated PBEsol Cu60 replicate C (solid-minus-liquid difference, 0.36 at.%). The largest absolute difference remained 0.85 at.%, and the sign varied among velocity replicates. No systematic Cu or Ag partitioning was detected on the approximately 90-100 ps coexistence timescale. This observation supports interpreting the calculations as compositionally constrained coexistence benchmarks, while not excluding slower equilibrium partitioning beyond the accessible trajectory length. The deliberately composition-mismatched calculations described below were therefore used to distinguish rapid chemical equilibration from retention of the imposed starting composition (Figure S4 and Table S6).

Composition-mismatched coexistence calculations. To distinguish a thermodynamic absence of partitioning from kinetic retention of the equal-composition initialization, additional PBEsol cells were prepared at overall compositions of 40, 50, and 60 at.% Cu with deliberately different Cu fractions in the initial upper solid and lower liquid regions. Starting from the existing 13,824-atom two-phase geometries, Cu and Ag type assignments were randomized separately within the two initial phase regions to create solid/liquid Cu pairs of 30/50, 20/60, and 10/70 at overall 40 at.% Cu; 40/60, 30/70, and 20/80 at overall 50 at.% Cu; and 70/50, 80/40, and 90/30 at overall 60 at.% Cu. We preserved the total atom count, global composition, atomic coordinates, cell dimensions, and initial interface geometry. The equal-composition 40/40, 50/50, and 60/60 baselines were not repeated because the primary coexistence calculations in Table S4 already represent them. Each perturbed cell was propagated with a 2 fs time step using three stages: 30 ps in which the initial-solid atoms were coupled to an NPT thermostat and barostat while the initial-liquid atoms evolved under the constant particle number, volume, and energy (NVE) ensemble, with the shared periodic cell dilated by the global barostat; 20 ps with independent NVT thermostats applied concurrently to the fixed initial-solid and initial-liquid atom groups; and 300 ps of whole-cell NPH dynamics at 1.01325 bar. We selected phase-specific thermostat targets from the PBEsol random-alloy coexistence-temperature curve to keep the designated solid below and the designated liquid above their composition-specific melting ranges as closely as possible. When the solid melted during preparation, we lowered only its thermostat target and reran the trajectory. The thermostat groups retain their initial atom IDs, so terminal phase compositions were evaluated spatially rather than from those fixed groups. Structural-order and Cu-composition profiles were accumulated in 50 bins along z every 5 ps. For each block, we normalized the local-order profile between its liquid and crystalline plateaus, identified the contiguous crystalline slab using a normalized-order threshold of 0.5, and evaluated bulk compositions from the central half of each structurally assigned phase region. All nine heterogeneous-composition conditions reached valid terminal two-phase states and are reported in Table S6. Each condition is represented by one completed interface-screened trajectory. We report mixed-trajectory temperatures and potential energies with autocorrelation-corrected standard errors from the final processing analysis, and define ΔT and ΔPE relative to the corresponding equal-composition benchmark. Because the trajectories

retain the imposed compositional mismatch, these quantities are sensitivity diagnostics rather than equilibrium liquidus or partitioning measurements.

Density and Enthalpy. We performed finite-temperature density and enthalpy simulations for Cu compositions of 0, 20, 40, 50, 60, 80, and 100 at.% over the available temperature range. The coexistence trends guided phase selection. Enthalpies were reported as H/N, with H = Etot + PV, and mixing enthalpies were referenced to pure Cu and pure Ag from the same functional, phase, and temperature. PBE mixing enthalpies use fully simulated phase-matched liquid endpoints throughout. For PBEsol, fully simulated phase-matched liquid mixing enthalpies are available at 1500 K, where all compositions and both endpoints remain liquid. At 1400 K, an approximate phase-matched PBEsol liquid mixing-enthalpy curve was additionally constructed using an explicitly estimated metastable pure-Cu liquid endpoint, as described below and reported in Table S9. No fully simulated phase-matched PBEsol liquid curve is reported at 1300 K because the pure-Cu liquid endpoint could not be maintained. The experimental density and mixing-enthalpy comparisons are 125-127 K lower than the 1500 K simulations. They are therefore used primarily to assess relative functional ordering, sign, and composition dependence rather than as strictly temperature-matched absolute benchmarks.

Finite-Temperature Ordered-Solid Mixing Enthalpies. Additional finite-temperature calculations were performed for ordered crystalline Cu–Ag configurations at 700 and 800 K using both the PBE- and PBEsol-trained potentials. The compositions were 0, 25, 50, 75, and 100 at.% Cu. Each simulation contained 6,912 atoms and was conducted in the NPT ensemble at approximately ambient pressure. The enthalpy per atom was evaluated as $H/N = (E_{tot} + PV)/N$. For each functional and temperature, the solid mixing enthalpy was calculated relative to the corresponding pure crystalline endpoints:

$$\Delta H_{mix}(x,T) = H(Cu_xAg_{1-x},T) - xH(Cu,T) - (1-x)H(Ag,T) \qquad (2)$$

We obtained the production enthalpies from the final half of the completed thermodynamic output (equation 2). We estimated statistical uncertainties from ten blocks and propagated them from the alloy and elemental reference enthalpies. All 20 simulations completed successfully, with no enthalpy-drift warnings. Visual inspection of the final configurations indicated that the ordered crystalline structures were retained. These calculations provide finite-temperature mixing enthalpies for the specified ordered configurations. They should not be interpreted as equilibrium free energies or as proof of their stability relative to the terminal solid solutions.

Self-Diffusion and Viscosity. Self-diffusion coefficients were obtained from 1000 ps constant particle number, volume, and energy (NVE) production trajectories using the long-time Einstein relation $D = (1/6)d\langle r^2\rangle/dt$. Candidate fitting windows required positive mean-square-displacement (MSD) slopes, a minimum MSD displacement of 2 Å², and $R^2 \geq 0.98$. Block-bootstrap uncertainties were calculated using 5 ps blocks, and replicate trajectories were averaged when available. We calculated viscosities using the Green-Kubo relation applied to the off-diagonal pressure-tensor autocorrelation functions. The workflow used NPT equilibration, NVT equilibration, NVE re-equilibration, and long NVE production trajectories. Stress autocorrelation functions were integrated and averaged over Pxy, Pxz, and Pyz; plateau selection and replicate scatter were propagated into the reported uncertainties. Figure S9

shows representative cumulative Green-Kubo integrals at 1475 K, with the final 20% of the 50 ps integration interval indicated as a convergence window. The PBEsol 1375 K pure-Cu point remains unavailable and is excluded from matched functional comparisons at 1375 K.

Neural-Network Potential Validation.

Table S1. Final validation RMSEs for the Cu-Ag neural-network potentials trained using PBE and PBEsol datasets. Cu_Ag-9-0/1/2 correspond to the PBE-trained model ensemble, while Cu_Ag-10-0/1/2 correspond to the PBEsol-trained ensemble. We report energy, force, and per-atom virial RMSEs in meV atom^{-1}, eV Å^{-1}, and eV atom^{-1}, respectively.

**Functional**	**Model**	**Energy RMSE (meV atom^{-1})**	**Force RMSE (eV Å^{-1})**	**Virial RMSE (eV atom^{-1})**
PBE	CuAg-9-0	2.24	0.0161	0.0164
PBE	CuAg-9-1	2.81	0.0229	0.00891
PBE	CuAg-9-2	1.83	0.0150	0.00803
**PBE**	**Mean ± SEM**	2.29 ± 0.28	0.0180 ± 0.0025	0.01111 ± 0.00266
PBEsol	CuAg-10-0	5.55	0.0217	0.0119
PBEsol	CuAg-10-1	4.58	0.0187	0.0111
PBEsol	CuAg-10-2	4.38	0.0239	0.0102

The mean PBE validation errors are 2.29 ± 0.28 meV atom^{-1} for energy, 0.0180 ± 0.0025 eV Å^{-1} for forces, and 0.01111 ± 0.00266 eV atom^{-1} for per-atom virials. The mean PBEsol validation errors are 4.84 ± 0.36 meV atom^{-1}, 0.02143 ± 0.00151 eV Å^{-1}, and 0.01107 ± 0.00049 eV atom^{-1}, respectively. These values are small relative to the functional-dependent EOS, cohesive-energy, melting-temperature, and liquid-transport shifts analyzed in the main text.

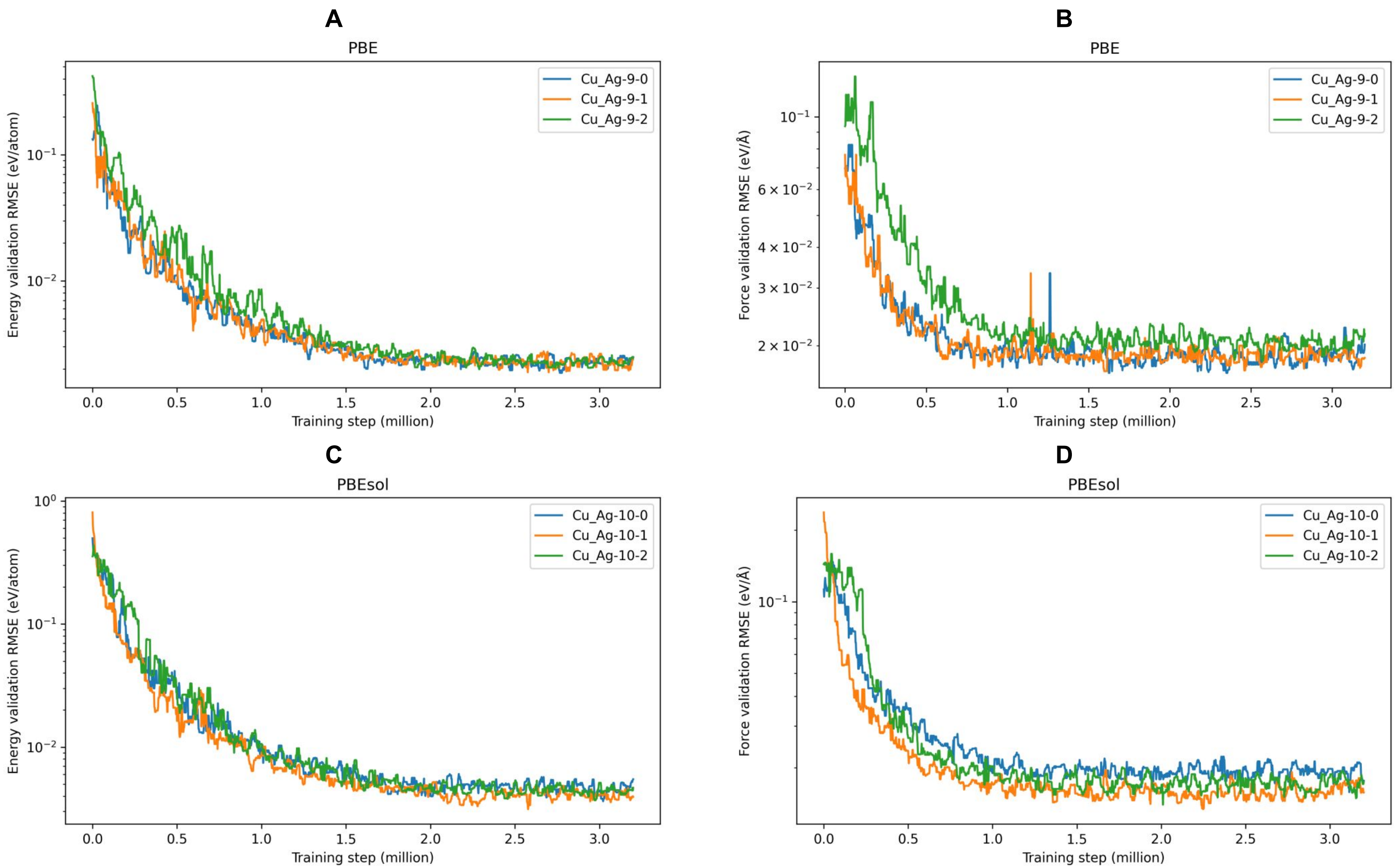

Figure S1. Validation-error learning curves for the independently seeded Cu-Ag model ensembles. (A,B) PBE energy and force validation RMSEs for Cu_Ag-9-0/1/2. (C,D) PBEsol energy and force validation RMSEs for Cu_Ag-10-0/1/2. Adaptive-learning configurations were selected from model-force disagreement and recomputed with the corresponding DFT functional.

Table S2. Comparison with selected machine-learned interatomic potentials developed and validated for metallic alloys. Values are reproduced using the normalization and validation definitions of the cited studies and are therefore not directly rank-comparable across rows.

Reference / system	Model	Validation data	Energy RMSE (meV atom^{-1})	Force RMSE (eV Å^{-1})	Virial / stress error	Finite-temperature or application validation
Present work, Cu-Ag (PBE)	DeepPot-SE	Held-out adaptive-learning validation; endpoint, alloy, solid, liquid, and interface systems	2.29 ± 0.28	0.0180 ± 0.0025	0.01111 ± 0.00266 eV atom^{-1}	TPC melting; density; diffusion; viscosity
Present work, Cu-Ag (PBEsol)	DeepPot-SE	Held-out adaptive-learning validation; endpoint, alloy, solid, liquid, and interface systems	4.84 ± 0.36	0.02143 ± 0.00151	0.01107 ± 0.00049 eV atom^{-1}	TPC melting; density; diffusion; viscosity
Rosenbrock et al. (2021), Ag-Pd [14]	SOAP-GAP	~6000 independent liquid configurations excluded from the active-learned training set	15.4	0.224	8.3 meV Å^{-3}	Independent liquid validation; GAP not used for the phase diagram
Rosenbrock et al. (2021), Ag-Pd [14]	MTP	~6000 independent liquid configurations excluded from the active-learned training set	10.9	0.241	12.7 meV Å^{-3}	Nested-sampling melting lines and alloy phase diagram
Byggmästar et al. (2022), Mo-Nb-Ta-V-W [15]	tabGAP (2b + 3b + EAM)	Separate crystalline and liquid test sets	~2-3 crystal; 12.4 liquid	Not tabulated numerically	Not reported	Solid-liquid-interface melting calculations
Marchand and Curtin (2022), Al-Cu-Mg-Zn [16]	Behler-Parrinello NNP	Held-out test structures; values reported before secondary focused training	32.00	0.06669	Not reported	Broad defect, interface, and metallurgical-property validation; no melting/transport test
Wang et al. (2020), Al-Mg [10]	Deep Potential	DFT-refined candidate alloy structures used for crystal-structure prediction	~2 total energy; ~6 formation energy	Not reported	Not reported	Crystal-structure search; no melting/transport test

The Rosenbrock virial entries are stress-like errors in meV Å^{-3}, whereas the present DeePMD virial metric is normalized per atom in eV atom^{-1}. The Byggmästar force errors were presented graphically rather than as a single numerical aggregate.

Table S3. (A) System-level coverage of the Cu-Ag DeepMD datasets used for training and validation. The accompanying summary files provide exact frame counts for each system; composition- and temperature-resolved frame totals are reported in Part B.

Functional	Split	Systems	Cu range (at.%)	Atoms/system	Encoded T range	Sources
PBE	Training	112	0-100	1-108	200-2200 K + unlabeled	Materials Project-derived; NO_MP
PBE	Validation	111	0-100	1-108	200-2200 K + unlabeled	Materials Project-derived; NO_MP
PBEsol	Training	103	0-100	1-108	200-2200 K + unlabeled	Materials Project-derived; NO_MP
PBEsol	Validation	98	0-100	2-108	200-2200 K + unlabeled	Materials Project-derived; NO_MP

Both functional-specific datasets span the pure-element endpoints and intermediate Cu-Ag compositions and contain Materials Project-derived and non-Materials-Project structures. Detailed configuration-level phase labels were not uniformly encoded in the archived paths. Temperature-unlabeled entries include static, relaxed, or otherwise non-MD structures. Part B reports the exact training and validation frame counts after filtering and deduplication, aggregated by nominal Cu composition and archived temperature block.

Table S3 (continued). (B) Training and validation frame counts by nominal Cu composition and archived temperature block. Entries are training frames (validation frames). A dash indicates no frames in that composition-temperature bin. “Unlabeled” denotes static, relaxed, or other configurations without an assigned temperature block. **PBE dataset: 29,638 training frames and 1,565 validation frames.**

**Cu (at.%)**	**200-400**	**600-800**	**800-1000**	**1200-1400**	**1400-1600**	**1800-2000**	**2000-2200**	Unknown	**Total**
0	2234 (117)	2273 (118)	—	1921 (102)	—	—	—	1879 (99)	**8307 (436)**
10	—	—	—	—	—	—	—	1909 (100)	**1909 (100)**
15	3 (1)	4 (1)	2 (1)	31 (2)	8 (1)	48 (2)	33 (2)	—	**129 (10)**
20	—	—	—	—	—	—	—	1304 (69)	**1304 (69)**
30	—	—	—	—	—	—	—	1540 (81)	**1540 (81)**
40	—	—	—	—	—	—	—	1372 (72)	**1372 (72)**
50	4 (1)	30 (2)	38 (2)	1 (0)	—	—	59 (3)	1158 (62)	**1290 (70)**
60	—	—	—	—	—	—	—	2013 (106)	**2013 (106)**
70	—	—	—	—	—	—	—	1929 (102)	**1929 (102)**
80	—	—	—	—	—	—	—	1732 (91)	**1732 (91)**
85	20 (1)	25 (1)	28 (1)	—	45 (2)	2 (1)	53 (3)	—	**173 (9)**
90	—	—	—	—	—	—	—	1700 (90)	**1700 (90)**
100	1445 (75)	1377 (73)	—	—	1290 (70)	—	—	2128 (111)	**6240 (329)**
**Total**	**3706 (195)**	**3709 (195)**	**68 (4)**	**1953 (104)**	**1343 (73)**	**50 (3)**	**145 (8)**	**18664 (983)**	**29638 (1565)**

PBEsol dataset: 31,614 training frames and 1,676 validation frames.

**Cu (at.%)**	**200-400**	**400-600**	**600-800**	**800-1000**	**1200-1400**	**1400-1600**	**1600-1800**	**1800-2000**	**2000-2200**	Unknown	**Total**
0	2931 (154)	1 (1)	3248 (171)	1 (1)	3561 (189)	—	1 (1)	—	—	8691 (458)	**18434 (975)**
10	—	—	—	—	—	—	—	—	—	25 (1)	**25 (1)**
20	70 (4)	—	85 (4)	71 (4)	81 (4)	87 (5)	—	—	—	623 (33)	**1017 (54)**
30	—	—	—	—	—	—	—	—	—	13 (1)	**13 (1)**
40	91 (5)	—	80 (4)	86 (5)	90 (5)	87 (5)	—	—	—	12 (1)	**446 (25)**
50	89 (5)	55 (3)	86 (4)	150 (8)	170 (9)	79 (4)	59 (3)	—	—	500 (27)	**1188 (63)**
60	96 (5)	23 (1)	95 (5)	124 (6)	125 (7)	102 (5)	24 (1)	—	—	23 (1)	**612 (31)**
70	—	16 (1)	—	15 (1)	14 (1)	—	11 (1)	—	—	65 (3)	**121 (7)**
80	81 (4)	69 (4)	94 (5)	149 (8)	148 (8)	92 (5)	68 (4)	—	—	22 (1)	**723 (39)**
85	—	—	—	—	—	—	—	5 (1)	—	—	**5 (1)**
90	—	14 (1)	—	6 (1)	39 (2)	—	8 (1)	—	—	24 (1)	**91 (6)**
100	—	1 (0)	—	1 (0)	1 (0)	—	1 (0)	—	35 (2)	8900 (471)	**8939 (473)**
**Total**	**3358 (177)**	**179 (11)**	**3688 (193)**	**603 (34)**	**4229 (225)**	**447 (24)**	**172 (11)**	**5 (1)**	**35 (2)**	**18898 (998)**	**31614 (1676)**

The PBE dataset contains 29,638 training and 1,565 validation frames, while the PBEsol dataset contains 31,614 training and 1,676 validation frames. Both datasets include intermediate nominal alloy compositions. The larger unlabeled contribution primarily reflects static, relaxed, and adaptive-learning configurations whose archived system names do not encode a temperature.

Table S3 (continued). (C) Final adaptive-learning ensemble-deviation tests across 11 Cu-Ag compositions. Each row summarizes 231 sampled frames (21 frames for each composition). Force deviations are reported in eV Å⁻¹.

**Functional**	**T (K)**	**Frames**	**⟨δF,avg⟩**	**Median δF,max**	**P95 δF,max**	**Maximum δF,max**	**Frames δF,max > 0.10**
PBE	400	231	0.0046	0.0108	0.0148	0.0174	0/231
PBE	800	231	0.0058	0.0138	0.0210	0.0285	0/231
PBE	1200	231	0.0081	0.0208	0.0350	0.0572	0/231
PBE	1600	231	0.0107	0.0304	0.0498	0.0918	0/231
PBEsol	400	231	0.0113	0.0232	0.0383	0.0576	0/231
PBEsol	800	231	0.0135	0.0290	0.0431	0.0688	0/231
PBEsol	1200	231	0.0162	0.0357	0.0546	0.0820	0/231
PBEsol	1600	231	0.0232	0.0559	0.0981	0.2740	11/231

Across 924 sampled frames per functional, the 95th percentile of δF,max was 0.0382 eV Å⁻¹ for PBE and 0.0689 eV Å⁻¹ for PBEsol. No PBE frame exceeded 0.10 eV Å⁻¹. Eleven PBEsol frames (1.2%) exceeded 0.10 eV Å⁻¹, all at 1600 K, and only three exceeded 0.15 eV Å⁻¹. The largest PBEsol value (0.2740 eV Å⁻¹) was an isolated high-temperature frame; the corresponding 1600 K trajectory-averaged atom-mean deviation was 0.0232 eV Å⁻¹.

Fixed-Composition Melting and Cohesive-Energy Diagnostics.

Table S4. Interface-screened replicate-level fixed-composition coexistence temperatures underlying Figure 1. Replicates A, B, and C use independent initial velocity seeds for the same composition and atomic occupancy. Retained entries are reported as T_{coex} ± the autocorrelation-corrected time-series standard error (K); the final column reports the mean ± SEM across retained velocity replicates. Five retained PBEsol trajectories ended at 45,000-47,000 NPH steps (90-94 ps) rather than the nominal 50,000-step endpoint but retained two spatially separated solid-liquid interfaces and were accepted. These statistical uncertainties quantify only terminal time-series sampling and initial-velocity replicate variability; they do not include systematic uncertainty from the exchange-correlation functional, MLIP construction, chemical occupancy, or the fixed-composition constraint.

Functional	Cu (at.%)	Class	Rep. A (K)	Rep. B (K)	Rep. C (K)	n	Mean ± SEM (K)
PBE	0	random	973.1 ± 1.6	977.9 ± 2.5	983.6 ± 5.0	3	978.2 ± 3.0
PBE	10	random	926.5 ± 3.3	915.3 ± 2.9	926.0 ± 1.4	3	922.6 ± 3.7
PBE	20	random	867.7 ± 1.8	873.9 ± 3.8	865.3 ± 3.7	3	869.0 ± 2.6
PBE	25	ordered	879.0 ± 1.3	880.5 ± 4.8	873.2 ± 2.2	3	877.6 ± 2.2
PBE	30	random	812.3 ± 1.5	813.2 ± 3.2	819.8 ± 2.6	3	815.1 ± 2.4
PBE	35	random	825.1 ± 3.3	811.5 ± 3.9	804.4 ± 3.5	3	813.7 ± 6.1
PBE	40	random	802.2 ± 4.7	801.5 ± 2.8	800.7 ± 2.8	3	801.4 ± 0.4
PBE	45	random	800.7 ± 4.2	798.5 ± 2.5	801.5 ± 4.4	3	800.2 ± 0.9
PBE	50	random	794.3 ± 3.3	798.3 ± 2.5	—	2	796.3 ± 2.0
PBE	50	ordered	792.2 ± 2.4	796.6 ± 3.2	791.2 ± 3.8	3	793.4 ± 1.7
PBE	55	random	805.5 ± 1.8	806.9 ± 1.9	810.3 ± 0.6	3	807.6 ± 1.4
PBE	60	random	823.3 ± 2.0	822.2 ± 1.4	818.7 ± 0.8	3	821.4 ± 1.4
PBE	65	random	846.7 ± 3.6	848.1 ± 1.5	850.3 ± 5.8	3	848.4 ± 1.0
PBE	70	random	882.2 ± 2.8	884.3 ± 3.8	878.0 ± 2.8	3	881.5 ± 1.9
PBE	75	ordered	947.5 ± 1.7	944.1 ± 1.9	946.5 ± 2.6	3	946.0 ± 1.0
PBE	80	random	956.0 ± 3.0	961.9 ± 4.9	962.8 ± 2.5	3	960.2 ± 2.1
PBE	90	random	1098.4 ± 1.5	1094.7 ± 0.8	1093.8 ± 0.9	3	1095.6 ± 1.4
PBE	100	random	1253.9 ± 1.5	1261.1 ± 1.8	1256.8 ± 1.9	3	1257.3 ± 2.1
PBEsol	0	random	1188.6 ± 3.5	1190.0 ± 4.6	1192.0 ± 1.5	3	1190.2 ± 1.0
PBEsol	10	random	1122.7 ± 6.3	1122.7 ± 1.1	1118.3 ± 3.0	3	1121.2 ± 1.5
PBEsol	20	random	1073.2 ± 4.4	1054.8 ± 5.1	1065.1 ± 6.8	3	1064.4 ± 5.3
PBEsol	25	ordered	1089.5 ± 3.9	1083.4 ± 5.0	1082.9 ± 1.9	3	1085.3 ± 2.1

Functional	Cu (at.%)	Class	Rep. A (K)	Rep. B (K)	Rep. C (K)	n	Mean ± SEM (K)
PBEsol	30	random	1009.2 ± 4.8	1012.7 ± 5.7	1009.4 ± 7.2	3	1010.4 ± 1.2
PBEsol	35	random	984.5 ± 4.4	983.7 ± 4.4	989.3 ± 6.7	3	985.8 ± 1.8
PBEsol	40	random	972.5 ± 4.5	979.4 ± 7.1	979.9 ± 1.9	3	977.3 ± 2.4
PBEsol	45	random	975.2 ± 6.7	968.5 ± 3.0	965.6 ± 5.6	3	969.8 ± 2.8
PBEsol	50	random	975.8 ± 3.7	Excluded†	966.8 ± 5.6	2	971.3 ± 4.5
PBEsol	50	ordered	986.6 ± 3.9	960.3 ± 3.4	965.2 ± 3.3	3	970.7 ± 8.1
PBEsol	55	random	980.8 ± 5.7	973.8 ± 2.7	977.8 ± 3.3	3	977.5 ± 2.0
PBEsol	60	random	988.1 ± 2.6	993.3 ± 2.5	1006.1 ± 6.9	3	995.8 ± 5.4
PBEsol	65	random	1002.8 ± 4.2	1011.5 ± 6.3	1012.1 ± 4.3	3	1008.8 ± 3.0
PBEsol	70	random	1044.6 ± 4.6	1032.7 ± 6.0	1050.7 ± 2.7	3	1042.7 ± 5.3
PBEsol	75	ordered	1099.4 ± 2.3	1087.8 ± 2.0	1090.6 ± 5.6	3	1092.6 ± 3.5
PBEsol	80	random	1117.8 ± 2.4	1120.0 ± 4.3	1111.5 ± 5.8	3	1116.4 ± 2.5
PBEsol	90	random	1248.5 ± 1.4	1250.3 ± 2.2	1245.3 ± 1.4	3	1248.0 ± 1.5
PBEsol	100	random	1403.3 ± 2.5	1396.7 ± 1.8	1404.4 ± 5.1	3	1401.5 ± 2.4

† PBEsol 50 at.% Cu random replicate B was excluded because the terminal z-resolved local-order profile became essentially uniform and the solid-liquid interface was lost. PBE 50 at.% Cu random replicate C was not present in the collected final dataset. All other listed trajectories were retained by spatial interface screening. The five accepted shorter PBEsol trajectories are 20 at.% random A, 50 at.% ordered A, 60 at.% random A, 75 at.% ordered A, and 80 at.% random A; these ended after 90-94 ps and retained clear two-phase terminal profiles.

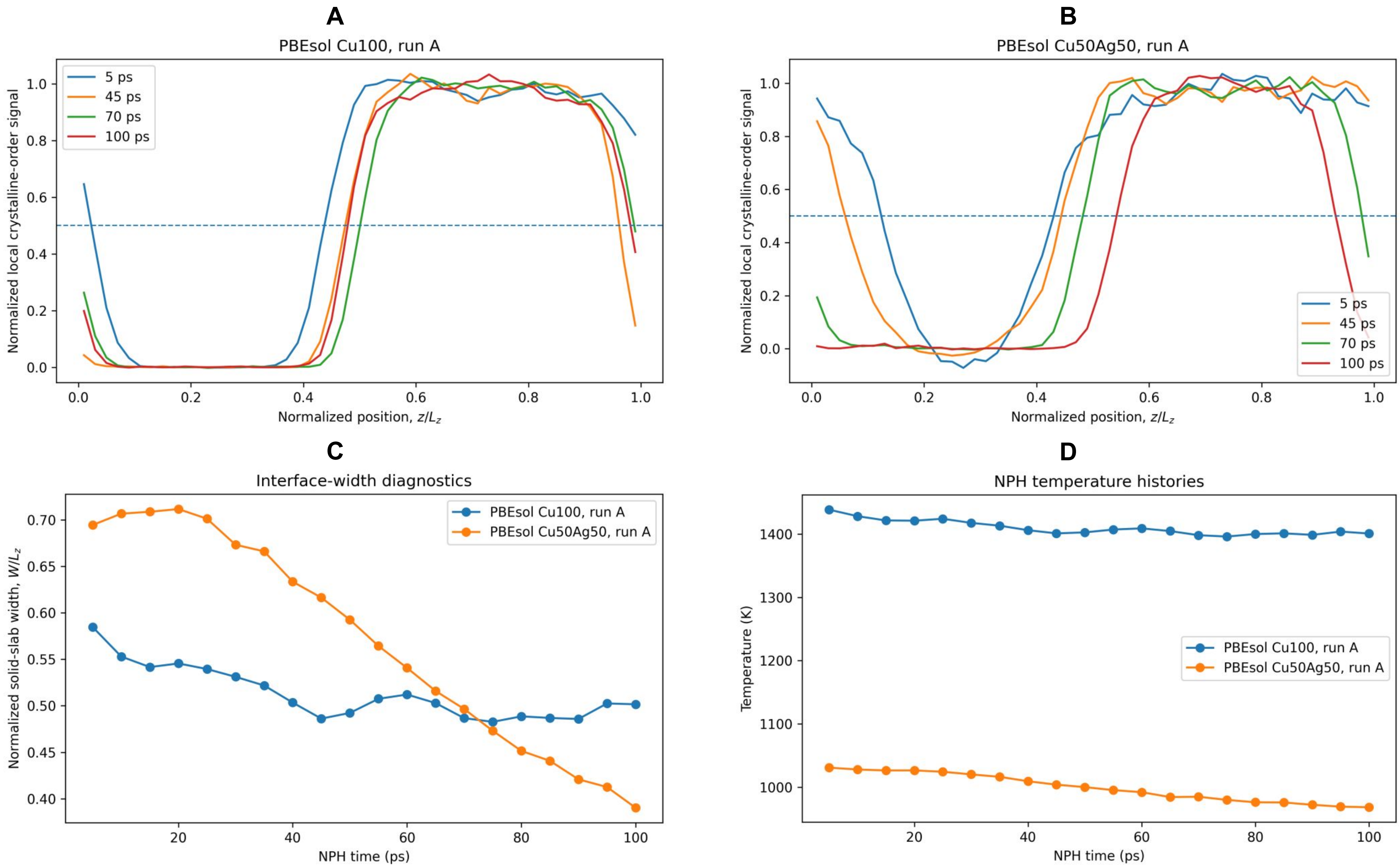

Figure S2. Representative spatial and thermodynamic diagnostics from PBEsol fixed-composition coexistence runs. (A) Normalized z-resolved crystalline-order profiles for Cu at 5, 45, 70, and 100 ps show two persistent interfaces and a stationary terminal slab width. (B) Corresponding profiles for $Cu_{50}Ag_{50}$ retain two interfaces while the slab translates and narrows during NPH latent-heat redistribution. (C) Normalized solid-slab widths and (D) NPH temperatures for the two examples. We used spatially resolved profiles, rather than the cell-averaged centrosymmetry count or an automated controller verdict, to identify retained two-phase trajectories.

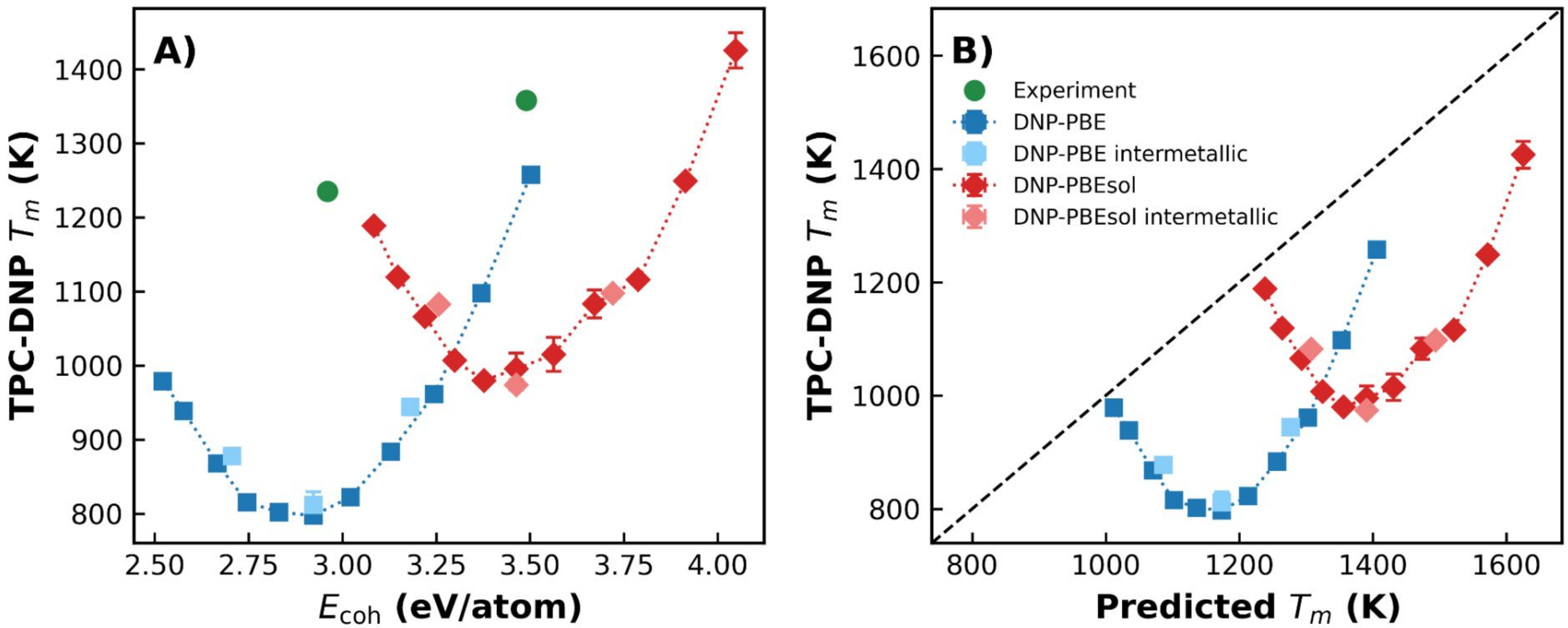

Figure S3. Cohesive energy and fixed-composition melting diagnostics. A) Coexistence temperature plotted against 0 K cohesive energy. B) Comparison with an empirical endpoint-based cohesive-energy prediction. Cohesive energy follows the endpoint ordering but does not account for the pronounced intermediate-composition melting depression.

Table S5. Endpoint-referenced melting-depression summary after spatial interface screening. The simulated maxima are evaluated over the sampled random-alloy compositions; the experimental value uses the assessed Cu-Ag eutectic and pure-element endpoints of Subramanian and Perepezko (1993). For the simulated values, the depression uncertainty is propagated from the replicate SEMs of the Ag endpoint, Cu endpoint, and composition at the sampled maximum as $\sigma\Delta = [(1-x)^2\sigma Ag^2 + x^2\sigma Cu^2 + \sigma T(x)^2]^{1/2}$. These uncertainties do not include systematic contributions from the functional, MLIP construction, chemical occupancy, or fixed-composition constraint. Because the endpoint-referenced depression is broad near its maximum and the composition grid is discrete, the sampled maxima at 55 at.% Cu for PBEsol and 60 at.% Cu for PBE should not be interpreted as precisely resolved equilibrium eutectic compositions.

Dataset	Ag endpoint (K)	Cu endpoint (K)	Cu at maximum depression (%)	T_{coex} at maximum depression (K)	Endpoint line (K)	Depression (K)	Basis
PBE	978.2	1257.3	60.0	821.4	1145.6	324.2 ± 2.2	maximum over screened random-alloy runs
PBEsol	1190.2	1401.5	55.0	977.5	1306.4	328.9 ± 2.5	maximum over screened random-alloy runs
Subramanian and Perepezko (1993)	1234.9	1357.8	39.9	1052.2	1283.9	231.7	assessed eutectic

Composition-Mismatched Coexistence Calculations. All nine PBEsol composition-mismatched calculations at overall compositions of 40, 50, and 60 at.% Cu retained valid spatially separated crystalline and liquid regions through the terminal 300 ps NPH stage. The terminal solid compositions changed by no more than 0.7 percentage points and the terminal liquid compositions by no more than 2.0 percentage points from their imposed values. Consequently, 97.4-103.4% of the initial solid-liquid composition mismatch was retained. Figure S4 shows four representative spatial profiles, while the complete nine-condition numerical results are reported in Table S6. Additional late-time spatial Cu-concentration profiles for the three 50 at.% overall-composition systems are provided in Figure S10.

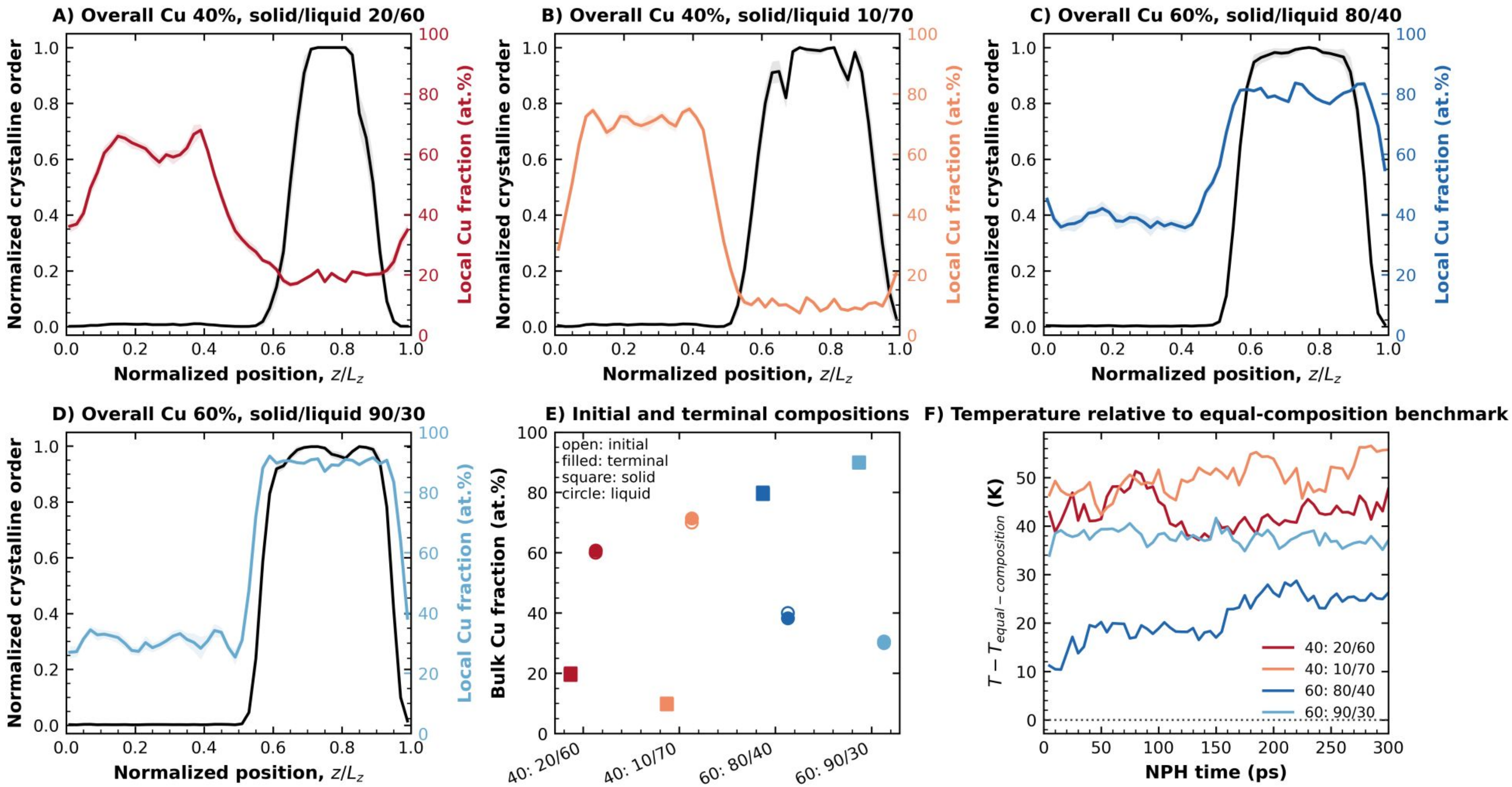

Figure S4. Representative PBEsol composition-mismatched coexistence trajectories. (A-D) Final-100-ps mean normalized crystalline-order and local Cu-composition profiles for four of the nine final conditions, initialized with solid/liquid Cu fractions of 20/60 and 10/70 at overall 40 at.% Cu and 80/40 and 90/30 at overall 60 at.% Cu; shaded bands show the standard deviation among 5 ps profile blocks. (E) Initial and terminal bulk solid and liquid Cu fractions for these representative trajectories. (F) NPH temperature histories relative to the campaign equal-composition reference values used when these heterogeneous calculations were analyzed, approximately 980 K at overall 40 at.% Cu and 1000 K at overall 60 at.% Cu. These plotted offsets are retained as run diagnostics; the numerical ΔT values in Table S6 are rebased to the final screened equal-composition benchmarks in Table S4. All four representative trajectories retained two interfaces and persistent composition differences through 300 ps. The complete nine-condition data are given in Table S6. The reported mixed-trajectory temperatures are preparation-dependent two-phase diagnostics and are not equilibrium liquidus points.

Table S6. Final PBEsol composition-mismatched coexistence results. All nine calculations have valid terminal two-phase states. Terminal solid/liquid compositions and their changes are referenced to the imposed starting values. Mismatch retained is the terminal $|x_{Cu,solid} - x_{Cu,liquid}|$ divided by the initial $|x_{Cu,solid} - x_{Cu,liquid}|$, expressed as a percentage. Standard T_{coex} values are the final screened equal-composition benchmarks from Table S4; mixed temperatures and potential energies are the final processed values reported for the heterogeneous trajectories. ΔT is mixed minus the final screened equal-composition T_{coex} and is reported to the nearest kelvin. The potential-energy columns retain the processed equal-composition energy references used in the original energy analysis, and ΔPE is mixed minus that standard PE. Displayed temperatures and energies are rounded. Each heterogeneous-composition condition is one completed interface-screened trajectory. These values quantify sensitivity to the prescribed composition pair and should not be interpreted as equilibrium liquidus temperatures or equilibrium partitioning. Composition-analysis distinction: the terminal solid/liquid Cu fractions in Table S6 are obtained using the structurally assigned bulk phase regions in the final processing analysis (central half of each phase region). Figure S10 instead reports late-time spatial-profile averages over the final 20% of the recorded NPH trajectory using fixed bulk windows. Consequently, these two diagnostics can yield slightly different bulk composition estimates for the same trajectory; the values should not be substituted for one another or interpreted as separate simulations.

**Overall Cu; initial solid/liquid (at.%)**	**Terminal solid/liquid Cu (at.%)**	**ΔCu solid/liquid (percentage points)**	**Mismatch retained (%)**	**Standard/mixed T (K)**	**ΔT (K)**	**Standard/mixed PE (eV atom⁻¹)**	**ΔPE (eV atom⁻¹)**
40; 30/50	30.2 / 50.0	0.2 / 0.0	99	977 ± 2 / 966 ± <1	-11	-3.437 ± 0.001 / -3.439 ± <0.001	-0.002
40; 20/60	19.4 / 60.0	-0.6 / 0.0	101.3	977 ± 2 / 1023 ± 1	+46	-3.437 ± 0.001 / -3.432 ± <0.001	+0.005
40; 10/70	9.6 / 71.4	-0.4 / 1.4	103	977 ± 2 / 1034 ± 1	+57	-3.437 ± 0.001 / -3.450 ± <0.001	-0.012
50; 40/60	40.7 / 60.1	0.7 / 0.1	97.4	971 ± 5 / 939 ± 1	-32	-3.528 ± 0.006 / -3.546 ± <0.001	-0.018
50; 30/70	29.9 / 70.9	-0.1 / 0.9	102.7	971 ± 5 / 942 ± 1	-29	-3.528 ± 0.006 / -3.549 ± <0.001	-0.021
50; 20/80	19.3 / 78.3	-0.7 / -1.7	98.3	971 ± 5 / 975 ± <1	+4	-3.528 ± 0.006 / -3.547 ± <0.001	-0.019
60; 70/50	70.2 / 49.6	0.2 / -0.4	102.9	996 ± 5 / 979 ± 1	-17	-3.634 ± 0.002 / -3.640 ± <0.001	-0.006
60; 80/40	79.3 / 38.0	-0.7 / -2.0	103.4	996 ± 5 / 1025 ± <1	+29	-3.634 ± 0.002 / -3.642 ± <0.001	-0.008
60; 90/30	89.8 / 30.8	-0.2 / 0.8	98.4	996 ± 5 / 1037 ± <1	+41	-3.634 ± 0.002 / -3.656 ± <0.001	-0.022

Across all nine completed trajectories, 97.4-103.4% of the imposed solid-liquid compositional mismatch is retained. The absolute terminal composition changes are at most 0.7 percentage points in the solid and 2.0 percentage points in the liquid, demonstrating persistent chemical memory on the 300 ps timescale rather than convergence toward a common partition. Using the final screened equal-composition benchmarks in Table S4, the coexistence-temperature shifts span -32 to +57 K: -11, +46, and +57 K for the three overall-40-at.% Cu conditions; -32, -29, and +4 K at overall 50 at.% Cu; and -17, +29, and +41 K at overall 60 at.% Cu. Thus, the sign and magnitude of the temperature response depend strongly on overall composition and on the prescribed solid-liquid pair; a given mismatch magnitude does not define a unique temperature shift across compositions. Potential-energy shifts range from -0.022 to +0.005 eV $atom^{-1}$ and do not uniquely track the temperature shifts. These trajectories therefore demonstrate kinetic retention of the imposed compositions but do not establish equality of component chemical potentials. Neither the mixed-trajectory temperature nor the potential-energy difference is an equilibrium liquidus criterion or a basis for ranking equilibrium partitions. Explicit chemical exchange, semi-grand-canonical sampling, or direct free-energy and chemical-potential calculations are required to determine equilibrium partitioning.

Equation-of-State and Surrogate Validation.

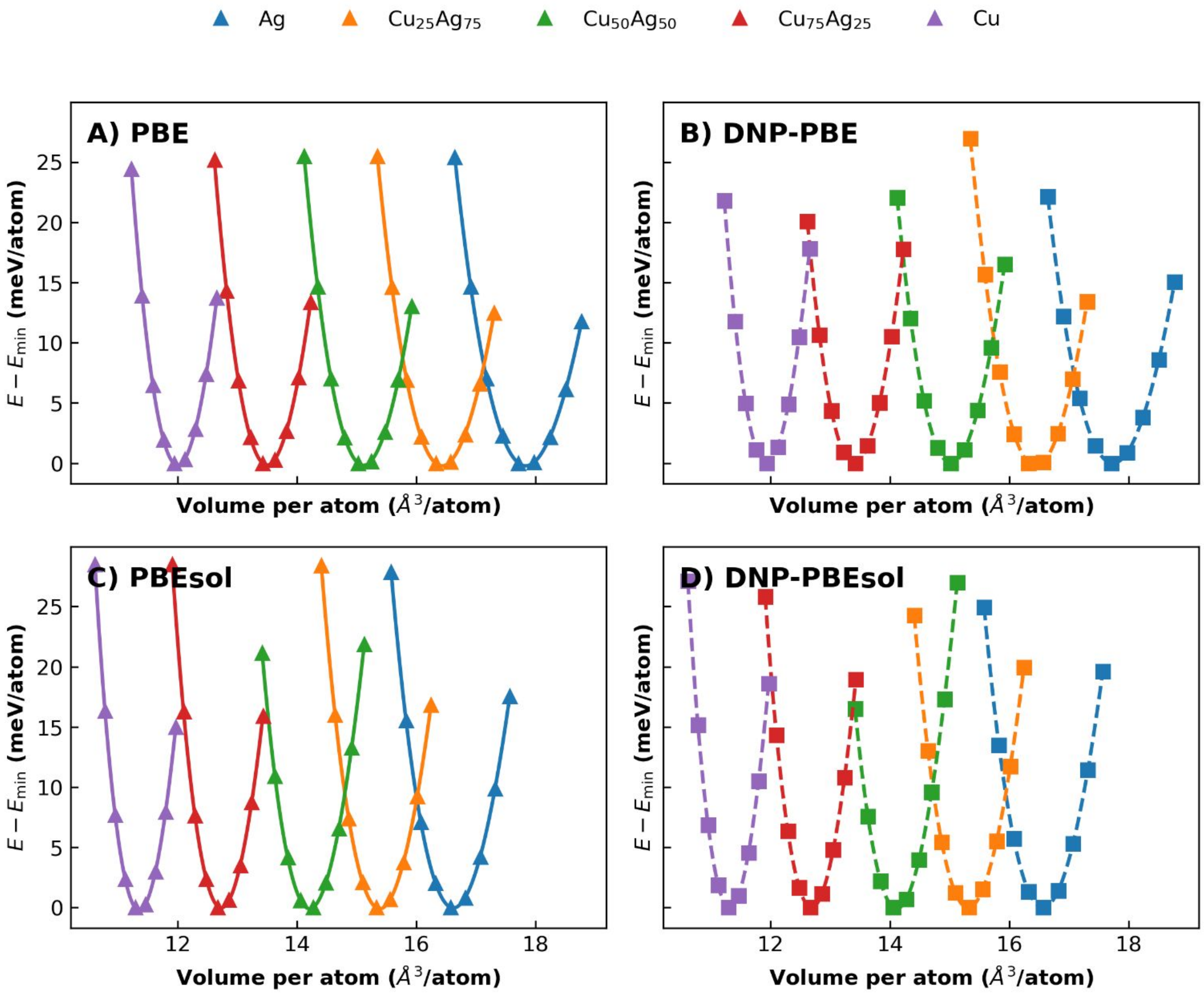

Figure S5. Equation-of-state behavior for Ag, $Cu_{25}Ag_{75}$, $Cu_{50}Ag_{50}$, $Cu_{75}Ag_{25}$, and Cu from PBE and PBEsol DFT and the corresponding DFT-surrogate models. Energies are referenced to the composition-specific minimum. PBEsol gives systematically smaller equilibrium volumes and stiffer energy-volume curves than PBE.

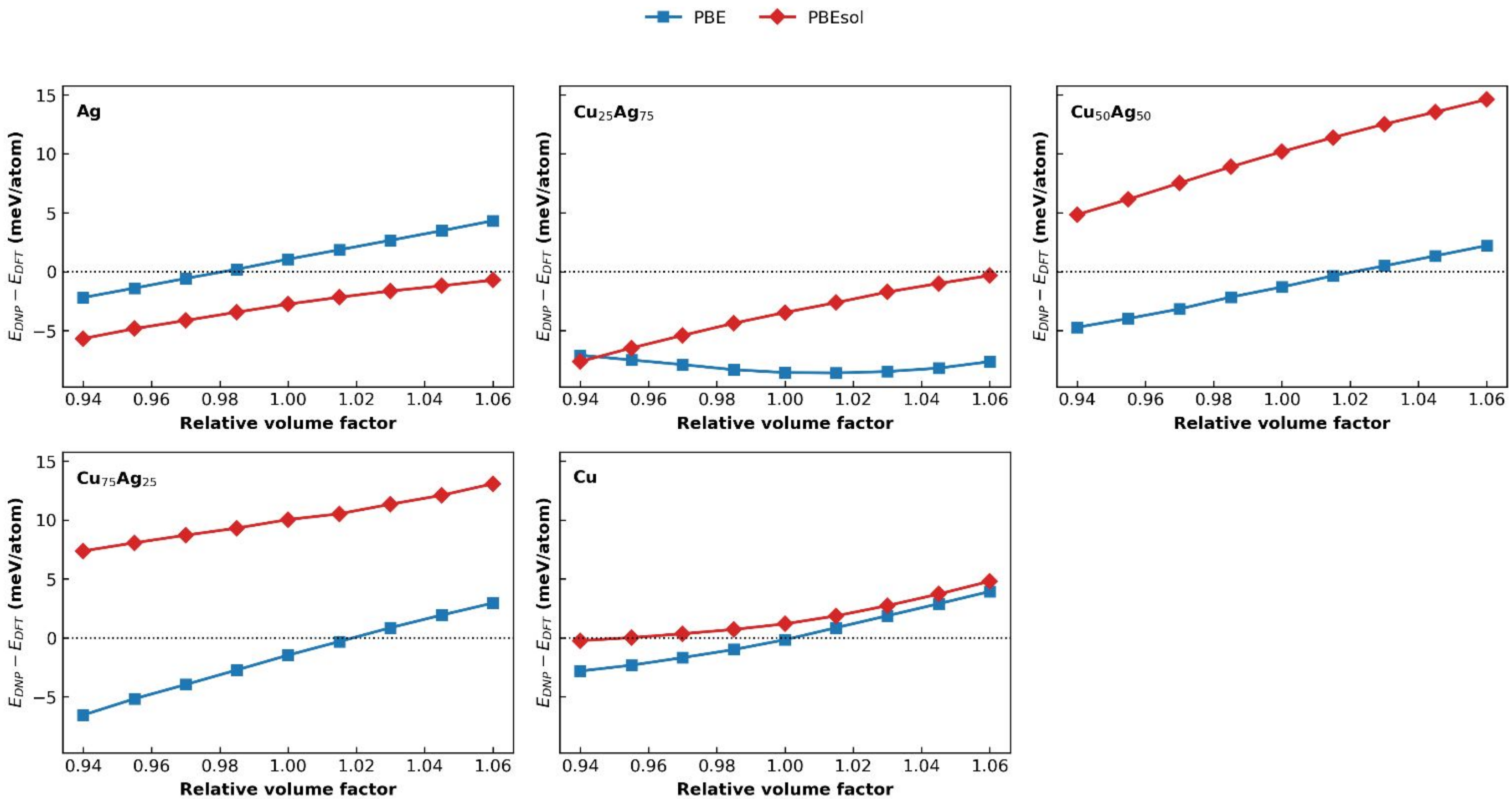

Figure S6. Pointwise surrogate-model energy deviations from the corresponding DFT EOS calculations for Ag, $Cu_{25}Ag_{75}$, $Cu_{50}Ag_{50}$, $Cu_{75}Ag_{25}$, and Cu. Positive values indicate that the surrogate energy is higher than the DFT reference.

Table S7. Corrected third-order Birch-Murnaghan equation-of-state fit parameters for DFT and DNP EOS curves. E_0 and V_0 are reported per atom; B0′ is dimensionless.

Functional	Composition	Method	E_0 (eV/atom)	V_0 (Å3/atom)	B_0 (GPa)	B_0'	Fit RMSE (meV/atom)
PBE	Ag	DFT	-2.717	17.8339	88.2	6.044	0.011
PBE	Ag	DNP	-2.716	17.7368	91.2	5.958	0.002
PBE	Cu25Ag75	DFT	-2.899	16.4305	98.5	5.908	0.023
PBE	Cu25Ag75	DNP	-2.908	16.4406	105.7	4.480	0.007
PBE	Cu50Ag50	DFT	-3.103	15.1136	109.2	5.316	0.039
PBE	Cu50Ag50	DNP	-3.104	15.0247	112.6	5.863	0.007
PBE	Cu75Ag25	DFT	-3.398	13.4986	122.6	5.140	0.034
PBE	Cu75Ag25	DNP	-3.399	13.3956	125.4	5.332	0.010
PBE	Cu	DFT	-3.731	12.0001	137.7	5.010	0.025
PBE	Cu	DNP	-3.731	11.9310	146.6	5.421	0.009
PBEsol	Ag	DFT	-3.310	16.6199	118.4	5.709	0.020
PBEsol	Ag	DNP	-3.312	16.5646	118.3	5.808	0.001
PBEsol	Cu25Ag75	DFT	-3.489	15.3862	127.0	5.592	0.024
PBEsol	Cu25Ag75	DNP	-3.492	15.3103	127.6	5.677	0.004
PBEsol	Cu50Ag50	DFT	-3.686	14.2108	135.7	5.322	0.019
PBEsol	Cu50Ag50	DNP	-3.676	14.1114	138.4	5.722	0.012
PBEsol	Cu75Ag25	DFT	-3.983	12.7297	149.4	5.574	0.049
PBEsol	Cu75Ag25	DNP	-3.972	12.6850	154.8	4.688	0.011
PBEsol	Cu	DFT	-4.311	11.3547	164.5	5.422	0.021
PBEsol	Cu	DNP	-4.310	11.3156	176.5	4.677	0.011

Note: EOS fits use the standard third-order Birch–Murnaghan form, including the factor $[6 - 4(V_0/V)^{2/3}]$.

Table S8. Pointwise EOS data used to generate Figures S5 and S6. DFT and DNP calculations use the same imposed cell volume at each volume factor. Absolute energies and composition-specific relative energies are reported per atom; ΔE is $E_{DNP} - E_{DFT}$. Relative energies and ΔE were calculated from unrounded energies; small differences may arise if recomputed from the displayed values.

Functional	Cu (at.%)	V/V_0	V (Å³ atom⁻¹)	E_{DFT} (eV atom⁻¹)	E_{DNP} (eV atom⁻¹)	E_{DFT}^{rel} (meV atom⁻¹)	E_{DNP}^{rel} (meV atom⁻¹)	$\Delta E_{DNP-DFT}$ (meV atom⁻¹)
PBE	0	0.940	16.646	-2.692	-2.694	25.38	22.13	-2.19
PBE	0	0.955	16.911	-2.703	-2.704	14.65	12.20	-1.39
PBE	0	0.970	17.177	-2.710	-2.711	7.04	5.40	-0.57
PBE	0	0.985	17.442	-2.715	-2.715	2.28	1.43	0.21
PBE	0	1.000	17.708	-2.717	-2.716	0.00	0.00	1.06
PBE	0	1.015	17.974	-2.717	-2.715	0.06	0.86	1.85
PBE	0	1.030	18.239	-2.715	-2.712	2.18	3.78	2.66
PBE	0	1.045	18.505	-2.711	-2.708	6.15	8.56	3.47
PBE	0	1.060	18.770	-2.705	-2.701	11.77	15.02	4.32
PBE	25	0.940	15.347	-2.874	-2.881	25.47	26.94	-7.08
PBE	25	0.955	15.592	-2.884	-2.892	14.60	15.67	-7.49
PBE	25	0.970	15.837	-2.892	-2.900	6.91	7.58	-7.88
PBE	25	0.985	16.082	-2.897	-2.905	2.19	2.43	-8.31
PBE	25	1.000	16.327	-2.899	-2.908	0.00	0.00	-8.55
PBE	25	1.015	16.572	-2.899	-2.907	0.11	0.08	-8.59
PBE	25	1.030	16.816	-2.897	-2.905	2.38	2.47	-8.46
PBE	25	1.045	17.061	-2.892	-2.901	6.61	6.98	-8.18
PBE	25	1.060	17.306	-2.886	-2.894	12.50	13.42	-7.63
PBE	50	0.940	14.120	-3.077	-3.082	25.47	22.05	-4.72
PBE	50	0.955	14.346	-3.088	-3.092	14.68	12.01	-3.97
PBE	50	0.970	14.571	-3.095	-3.099	7.04	5.18	-3.16
PBE	50	0.985	14.796	-3.100	-3.102	2.12	1.26	-2.16
PBE	50	1.000	15.022	-3.102	-3.104	0.00	0.00	-1.30

Functional	Cu (at.%)	V/V_0	V ($Å^3$ atom^{-1})	E_{DFT} (eV atom^{-1})	E_{DNP} (eV atom^{-1})	E_{DFT}^{rel} (meV atom^{-1})	E_{DNP}^{rel} (meV atom^{-1})	$\Delta E_{DNP-DFT}$ (meV atom^{-1})
PBE	50	1.015	15.247	-3.102	-3.103	0.17	1.12	-0.35
PBE	50	1.030	15.472	-3.100	-3.099	2.60	4.40	0.49
PBE	50	1.045	15.698	-3.095	-3.094	6.95	9.60	1.34
PBE	50	1.060	15.923	-3.089	-3.087	13.01	16.53	2.21
PBE	75	0.940	12.617	-3.372	-3.379	25.17	20.09	-6.54
PBE	75	0.955	12.818	-3.383	-3.388	14.35	10.65	-5.16
PBE	75	0.970	13.019	-3.391	-3.395	6.85	4.35	-3.96
PBE	75	0.985	13.221	-3.395	-3.398	2.15	0.89	-2.72
PBE	75	1.000	13.422	-3.398	-3.399	0.00	0.00	-1.46
PBE	75	1.015	13.623	-3.397	-3.398	0.28	1.43	-0.31
PBE	75	1.030	13.825	-3.395	-3.394	2.67	4.99	0.86
PBE	75	1.045	14.026	-3.391	-3.389	7.10	10.50	1.94
PBE	75	1.060	14.227	-3.384	-3.381	13.37	17.78	2.95
PBE	100	0.940	11.225	-3.706	-3.709	24.46	21.80	-2.81
PBE	100	0.955	11.404	-3.716	-3.719	13.92	11.77	-2.32
PBE	100	0.970	11.583	-3.724	-3.726	6.48	4.97	-1.67
PBE	100	0.985	11.762	-3.728	-3.729	1.96	1.13	-0.99
PBE	100	1.000	11.941	-3.730	-3.731	0.00	0.00	-0.16
PBE	100	1.015	12.120	-3.730	-3.729	0.31	1.33	0.86
PBE	100	1.030	12.299	-3.728	-3.726	2.85	4.89	1.88
PBE	100	1.045	12.478	-3.723	-3.720	7.40	10.45	2.89
PBE	100	1.060	12.657	-3.717	-3.713	13.73	17.82	3.93
PBEsol	0	0.940	15.578	-3.282	-3.287	27.86	24.94	-5.67
PBEsol	0	0.955	15.827	-3.294	-3.299	15.54	13.46	-4.84
PBEsol	0	0.970	16.075	-3.302	-3.307	7.10	5.71	-4.14
PBEsol	0	0.985	16.324	-3.308	-3.311	2.01	1.33	-3.43
PBEsol	0	1.000	16.572	-3.310	-3.312	0.00	0.00	-2.75
PBEsol	0	1.015	16.821	-3.309	-3.311	0.83	1.41	-2.17
PBEsol	0	1.030	17.070	-3.305	-3.307	4.19	5.31	-1.64

Functional	Cu (at.%)	V/V_0	V ($Å^3$ atom^{-1})	E_{DFT} (eV atom^{-1})	E_{DNP} (eV atom^{-1})	E_{DFT}^{rel} (meV atom^{-1})	E_{DNP}^{rel} (meV atom^{-1})	$\Delta E_{DNP-DFT}$ (meV atom^{-1})
PBEsol	0	1.045	17.318	-3.300	-3.301	9.89	11.44	-1.20
PBEsol	0	1.060	17.567	-3.292	-3.293	17.56	19.60	-0.71
PBEsol	25	0.940	14.407	-3.460	-3.468	28.43	24.27	-7.62
PBEsol	25	0.955	14.637	-3.473	-3.479	16.02	13.00	-6.47
PBEsol	25	0.970	14.867	-3.481	-3.487	7.39	5.44	-5.40
PBEsol	25	0.985	15.097	-3.487	-3.491	2.13	1.21	-4.37
PBEsol	25	1.000	15.327	-3.489	-3.492	0.00	0.00	-3.46
PBEsol	25	1.015	15.557	-3.488	-3.491	0.68	1.51	-2.62
PBEsol	25	1.030	15.787	-3.485	-3.487	3.76	5.50	-1.72
PBEsol	25	1.045	16.017	-3.479	-3.480	9.25	11.71	-1.00
PBEsol	25	1.060	16.246	-3.472	-3.472	16.83	19.96	-0.33
PBEsol	50	0.940	13.416	-3.664	-3.660	21.14	16.49	4.84
PBEsol	50	0.955	13.630	-3.675	-3.668	10.91	7.55	6.14
PBEsol	50	0.970	13.844	-3.681	-3.674	4.15	2.18	7.52
PBEsol	50	0.985	14.058	-3.685	-3.676	0.60	0.00	8.89
PBEsol	50	1.000	14.272	-3.686	-3.675	0.00	0.69	10.18
PBEsol	50	1.015	14.486	-3.683	-3.672	2.07	3.96	11.38
PBEsol	50	1.030	14.700	-3.679	-3.666	6.55	9.58	12.51
PBEsol	50	1.045	14.914	-3.672	-3.659	13.26	17.32	13.54
PBEsol	50	1.060	15.129	-3.664	-3.649	21.88	27.00	14.61
PBEsol	75	0.940	11.909	-3.954	-3.947	28.48	25.82	7.37
PBEsol	75	0.955	12.099	-3.966	-3.958	16.28	14.31	8.06
PBEsol	75	0.970	12.289	-3.975	-3.966	7.67	6.34	8.71
PBEsol	75	0.985	12.479	-3.980	-3.971	2.38	1.65	9.31
PBEsol	75	1.000	12.669	-3.982	-3.972	0.00	0.00	10.03
PBEsol	75	1.015	12.859	-3.982	-3.971	0.64	1.13	10.52
PBEsol	75	1.030	13.049	-3.979	-3.968	3.51	4.81	11.34
PBEsol	75	1.045	13.240	-3.974	-3.962	8.76	10.81	12.09
PBEsol	75	1.060	13.430	-3.967	-3.954	15.90	18.94	13.07

Functional	Cu (at.%)	V/V_0	V (Å^3 atom^{-1})	E_{DFT} (eV atom^{-1})	E_{DNP} (eV atom^{-1})	E_{DFT}^{rel} (meV atom^{-1})	E_{DNP}^{rel} (meV atom^{-1})	$\Delta E_{DNP-DFT}$ (meV atom^{-1})
PBEsol	100	0.940	10.613	-4.283	-4.283	28.52	27.09	-0.24
PBEsol	100	0.955	10.782	-4.295	-4.295	16.33	15.16	0.02
PBEsol	100	0.970	10.952	-4.303	-4.303	7.68	6.84	0.35
PBEsol	100	0.985	11.121	-4.309	-4.308	2.35	1.87	0.72
PBEsol	100	1.000	11.291	-4.311	-4.310	0.00	0.00	1.19
PBEsol	100	1.015	11.460	-4.311	-4.309	0.29	0.97	1.87
PBEsol	100	1.030	11.629	-4.308	-4.305	2.99	4.54	2.73
PBEsol	100	1.045	11.799	-4.303	-4.299	7.96	10.49	3.71
PBEsol	100	1.060	11.968	-4.296	-4.291	14.99	18.60	4.80

Thermophysical and Transport Diagnostics.

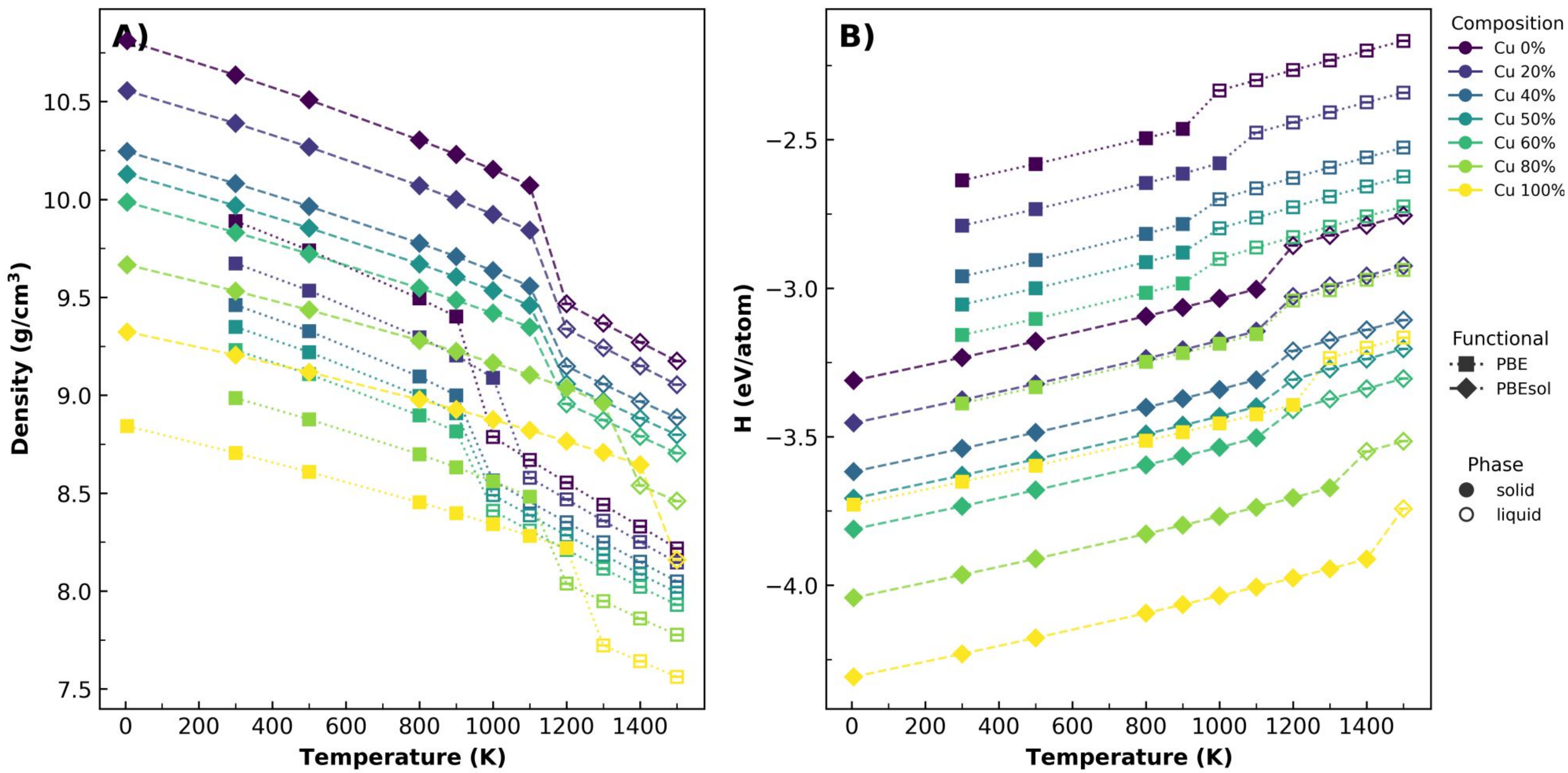

Figure S7. Temperature-dependent density and enthalpy trends for Cu-Ag. A) Density versus temperature for selected compositions, functionals, and phases. B) Enthalpy per atom versus temperature. PBEsol maintains the higher-density/lower-volume trend over temperature, and solid-liquid transitions produce the largest discontinuities in the finite-temperature response.

Table S9. Selected finite-temperature density and enthalpy data at 1300-1500 K. Uncertainties for simulated entries are SEMs from the analyzed trajectory blocks. PBE mixing enthalpies use phase-matched liquid endpoints throughout. PBEsol values at 1500 K are fully simulated and phase-matched. At 1400 K, the pure-Cu liquid endpoint was estimated from the liquid thermal increments across 0-80 at.% Cu and used to construct an approximate phase-matched PBEsol mixing-enthalpy curve.

Functional	T (K)	Cu (at.%)	Phase	Density ± SEM (g/cm3)	H ± SEM (eV/atom)	ΔH_{mix} ± SEM (meV/atom)	Ag/Cu refs.
PBE	1300	0	liquid	8.4403 ± 0.0009	-2.2329 ± 0.0003	0.0 ± 0.4	L/L
PBE	1300	20	liquid	8.3588 ± 0.0009	-2.4080 ± 0.0003	25.3 ± 0.3	L/L
PBE	1300	40	liquid	8.2492 ± 0.0008	-2.5938 ± 0.0003	40.0 ± 0.4	L/L
PBE	1300	50	liquid	8.1849 ± 0.0008	-2.6914 ± 0.0003	42.7 ± 0.4	L/L
PBE	1300	60	liquid	8.1141 ± 0.0008	-2.7928 ± 0.0003	41.5 ± 0.3	L/L
PBE	1300	80	liquid	7.9482 ± 0.0007	-3.0065 ± 0.0003	28.3 ± 0.4	L/L
PBE	1300	100	liquid	7.7221 ± 0.0006	-3.2353 ± 0.0003	0.0 ± 0.4	L/L
PBE	1400	0	liquid	8.3293 ± 0.0011	-2.2000 ± 0.0003	0.0 ± 0.4	L/L
PBE	1400	20	liquid	8.2504 ± 0.0010	-2.3745 ± 0.0003	25.6 ± 0.4	L/L
PBE	1400	40	liquid	8.1489 ± 0.0008	-2.5598 ± 0.0003	40.4 ± 0.3	L/L
PBE	1400	50	liquid	8.0867 ± 0.0008	-2.6575 ± 0.0003	42.8 ± 0.4	L/L
PBE	1400	60	liquid	8.0210 ± 0.0008	-2.7579 ± 0.0003	42.4 ± 0.3	L/L
PBE	1400	80	liquid	7.8593 ± 0.0007	-2.9716 ± 0.0003	28.8 ± 0.4	L/L
PBE	1400	100	liquid	7.6415 ± 0.0006	-3.2005 ± 0.0003	0.0 ± 0.4	L/L
PBE	1500	0	liquid	8.2177 ± 0.0009	-2.1675 ± 0.0003	0.0 ± 0.4	L/L
PBE	1500	20	liquid	8.1455 ± 0.0011	-2.3421 ± 0.0003	25.2 ± 0.4	L/L
PBE	1500	40	liquid	8.0488 ± 0.0009	-2.5268 ± 0.0003	40.4 ± 0.4	L/L
PBE	1500	50	liquid	7.9921 ± 0.0009	-2.6243 ± 0.0003	42.7 ± 0.3	L/L
PBE	1500	60	liquid	7.9295 ± 0.0008	-2.7254 ± 0.0003	41.6 ± 0.3	L/L
PBE	1500	80	liquid	7.7764 ± 0.0007	-2.9385 ± 0.0003	28.4 ± 0.4	L/L
PBE	1500	100	liquid	7.5618 ± 0.0005	-3.1666 ± 0.0003	0.0 ± 0.4	L/L
PBEsol	1300	0	liquid	9.3676 ± 0.0007	-2.8224 ± 0.0003	—	-

Functional	T (K)	Cu (at.%)	Phase	Density ± SEM (g/cm3)	H ± SEM (eV/atom)	ΔH_{mix} ± SEM (meV/atom)	Ag/Cu refs.
PBEsol	1300	20	liquid	9.2432 ± 0.0007	-2.9922 ± 0.0003	—	-
PBEsol	1300	40	liquid	9.0567 ± 0.0006	-3.1751 ± 0.0003	—	-
PBEsol	1300	50	liquid	8.9694 ± 0.0007	-3.2724 ± 0.0003	—	-
PBEsol	1300	60	liquid	8.8728 ± 0.0007	-3.3736 ± 0.0003	—	-
PBEsol	1300	80	solid	8.9608 ± 0.0005	-3.6721 ± 0.0002	—	-
PBEsol	1300	100	solid	8.7073 ± 0.0003	-3.9448 ± 0.0002	—	-
PBEsol	1400	0	liquid	9.2701 ± 0.0008	-2.7888 ± 0.0003	0.0 ± 0.4	L/L*
PBEsol	1400	20	liquid	9.1492 ± 0.0007	-2.9588 ± 0.0003	27.5 ± 0.4	L/L*
PBEsol	1400	40	liquid	8.9684 ± 0.0007	-3.1404 ± 0.0003	43.4 ± 0.5	L/L*
PBEsol	1400	50	liquid	8.8822 ± 0.0007	-3.2387 ± 0.0003	43.8 ± 0.5	L/L*
PBEsol	1400	60	liquid	8.7896 ± 0.0007	-3.3384 ± 0.0003	43.0 ± 0.6	L/L*
PBEsol	1400	80	liquid	8.5392 ± 0.0006	-3.5498 ± 0.0003	29.0 ± 0.7	L/L*
PBEsol	1400	100	solid	8.6454 ± 0.0003	-3.9123 ± 0.0002	—	-
PBEsol	1400	100	liquid (estimated)*	8.2339 ± 0.0050	-3.7763 ± 0.0008	0.0 (defined)	L/L*
PBEsol	1500	0	liquid	9.1746 ± 0.0008	-2.7553 ± 0.0003	0.0 ± 0.5	L/L
PBEsol	1500	20	liquid	9.0540 ± 0.0009	-2.9248 ± 0.0003	28.0 ± 0.4	L/L
PBEsol	1500	40	liquid	8.8857 ± 0.0008	-3.1075 ± 0.0003	42.7 ± 0.4	L/L
PBEsol	1500	50	liquid	8.7983 ± 0.0007	-3.2045 ± 0.0003	44.4 ± 0.4	L/L
PBEsol	1500	60	liquid	8.7041 ± 0.0007	-3.3042 ± 0.0003	43.4 ± 0.4	L/L
PBEsol	1500	80	liquid	8.4594 ± 0.0007	-3.5152 ± 0.0003	29.8 ± 0.4	L/L
PBEsol	1500	100	liquid	8.1590 ± 0.0005	-3.7424 ± 0.0003	0.0 ± 0.5	L/L

At 1400 K, directly simulated pure PBEsol Cu crystallized; the observed solid entry is retained in Table S9. The separate metastable-liquid Cu estimates used for Figure 4A (density) and Figure 3B (mixing enthalpy) are $\rho = 8.234 \pm 0.005$ g cm^{-3} and H = -3.7763 ± 0.0008 eV atom^{-1}, respectively. The density was obtained by linearly extrapolating the 1400-1500 K liquid-density increment with composition to 100 at.% Cu; the enthalpy was obtained from the mean liquid enthalpy increment over 0-80 at.% Cu. Estimated entries are explicitly labeled and are not treated as independent simulation trajectories.

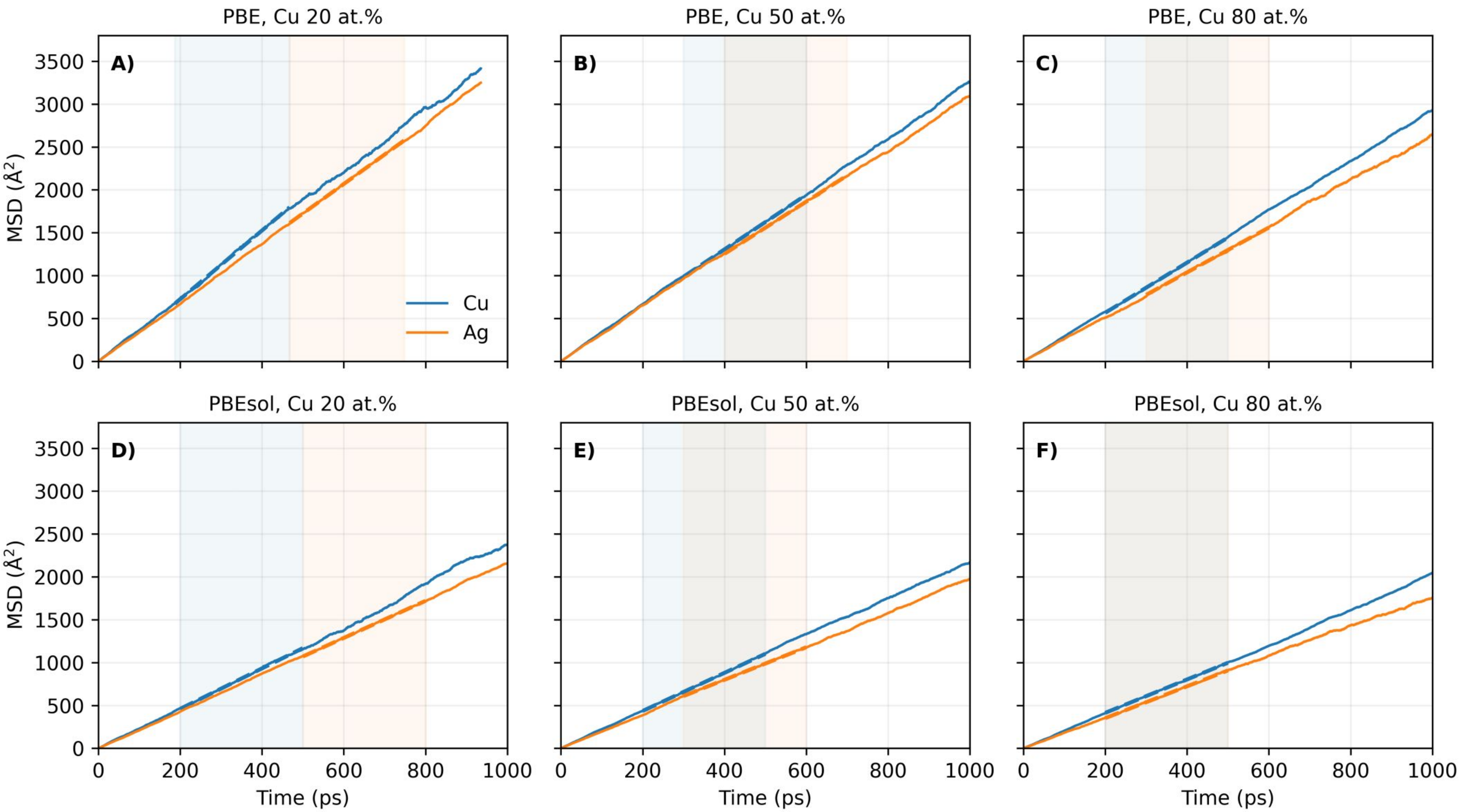

Figure S8. Representative Cu and Ag mean-squared-displacement (MSD) diagnostics at 1423 K for Ag-rich (Cu 20 at.%), intermediate (Cu 50 at.%), and Cu-rich (Cu 80 at.%) liquids using (A-C) PBE and (D-F) PBEsol. Solid curves show the 1000 ps NVE MSD trajectories for replicate 01, and dashed segments show the independently selected long-time linear fitting windows used in the Einstein relation. The lightly shaded intervals indicate the corresponding species-specific fitting windows. All displayed fits have $R^2 \geq 0.9989$ and MSD displacements substantially greater than the 2 Å² acceptance threshold.

Table S10. Composition-dependent self-diffusion coefficients at 1423 and 1573 K. Values are aggregate means ± replicate SEM in units of 10^{-9} m^2 s^{-1}. A dash denotes species absent at a pure-element endpoint.

Functional	T (K)	Cu (at.%)	D(Cu) ± SEM	n(Cu)	R^2(Cu)	D(Ag) ± SEM	n(Ag)	R^2(Ag)
PBE	1423	0	—	0	—	6.188 ± 0.124	3	0.9996
PBE	1423	20	6.727 ± 0.054	3	0.9988	5.704 ± 0.067	3	0.9997
PBE	1423	40	5.654 ± 0.090	3	0.9993	5.290 ± 0.097	3	0.9995
PBE	1423	50	5.577 ± 0.166	3	0.9996	5.357 ± 0.138	3	0.9995
PBE	1423	60	5.175 ± 0.203	3	0.9996	4.574 ± 0.116	3	0.9993
PBE	1423	80	4.798 ± 0.052	3	0.9997	4.369 ± 0.027	3	0.9991
PBE	1423	100	4.177 ± 0.054	3	0.9996	—	0	—
PBE	1573	0	—	0	—	7.240 ± 0.108	3	0.9996
PBE	1573	20	7.556 ± 0.220	3	0.9984	7.036 ± 0.080	3	0.9996
PBE	1573	40	7.342 ± 0.287	3	0.9995	6.575 ± 0.130	3	0.9997
PBE	1573	50	6.803 ± 0.145	3	0.9994	6.607 ± 0.213	3	0.9995
PBE	1573	60	6.921 ± 0.094	3	0.9996	6.512 ± 0.064	3	0.9993
PBE	1573	80	6.058 ± 0.190	3	0.9998	5.336 ± 0.324	3	0.9982
PBE	1573	100	5.747 ± 0.052	2	0.9998	—	0	—
PBEsol	1423	0	—	0	—	3.757 ± 0.009	3	0.9998
PBEsol	1423	20	4.022 ± 0.085	2	0.9991	3.672 ± 0.067	2	0.9996
PBEsol	1423	40	3.884 ± 0.104	3	0.9994	3.488 ± 0.095	3	0.9997
PBEsol	1423	50	3.798 ± 0.048	3	0.9996	3.294 ± 0.069	3	0.9996
PBEsol	1423	60	3.534 ± 0.209	3	0.9995	3.019 ± 0.078	3	0.9991
PBEsol	1423	80	3.305 ± 0.037	3	0.9997	3.005 ± 0.043	3	0.9993
PBEsol	1423	100	3.053 ± 0.001	2	0.9998	—	0	—
PBEsol	1573	0	—	0	—	4.916 ± 0.118	3	0.9997
PBEsol	1573	20	4.896 ± 0.149	3	0.9987	4.765 ± 0.029	3	0.9997
PBEsol	1573	40	5.105 ± 0.127	3	0.9994	4.809 ± 0.146	3	0.9995
PBEsol	1573	50	4.757 ± 0.122	3	0.9994	4.271 ± 0.024	3	0.9996
PBEsol	1573	60	4.726 ± 0.097	3	0.9998	4.258 ± 0.032	3	0.9991

Functional	T (K)	Cu (at.%)	D(Cu) ± SEM	n(Cu)	R^2(Cu)	D(Ag) ± SEM	n(Ag)	R^2(Ag)
PBEsol	1573	80	4.429 ± 0.105	3	0.9996	3.891 ± 0.067	3	0.9988
PBEsol	1573	100	4.031 ± 0.162	3	0.9997	—	0	—

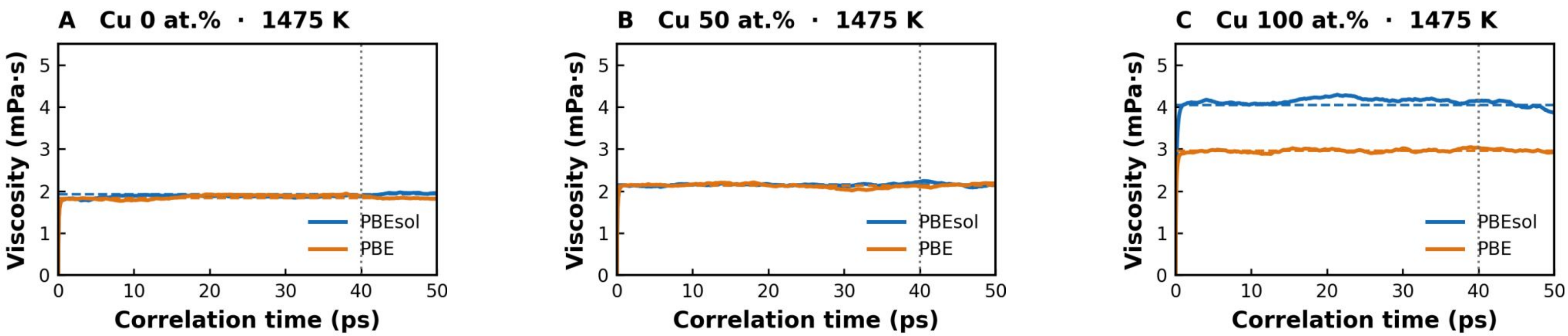

Figure S9. Representative cumulative Green–Kubo viscosity integrals at 1475 K for (A) Cu 0 at.%, (B) Cu 50 at.%, and (C) Cu 100 at.%. Solid curves show the replicate-averaged cumulative viscosity integrals from three independent trajectories for each functional. Horizontal dashed lines indicate the corresponding aggregate viscosities obtained by averaging each replicate integral over the final 20% (40–50 ps) of the correlation window, as reported in Table S11. The vertical dotted line at 40 ps marks the beginning of this averaging interval. The instantaneous 50 ps values need not equal the tail-averaged values.

Table S11. Composition-dependent viscosities of liquid Cu-Ag obtained from Green-Kubo stress-autocorrelation analysis. Simulated values are the mean ± SEM across three independent replicate trajectories and are reported in mPa·s. The component coefficient of variation (CV) is the replicate-averaged CV among the P_{xy}, P_{xz}, and P_{yz} viscosity estimates and is included as a convergence diagnostic. Δη is η(PBEsol) - η(PBE) and is reported only for matched functional comparisons. The PBEsol 1375 K pure-Cu point was not included in the matched analysis.

T (K)	Cu (at.%)	PBE viscosity ± SEM (mPa·s)	PBE component CV (%)	PBEsol viscosity ± SEM (mPa·s)	PBEsol component CV (%)	Δη (mPa·s)
1375	0	2.046 ± 0.061	12.9	3.454 ± 0.128	15.7	1.408
1375	20	2.205 ± 0.055	10.7	3.422 ± 0.185	20.6	1.216
1375	40	2.641 ± 0.044	15.3	3.520 ± 0.192	8.1	0.879
1375	50	2.663 ± 0.369	17.4	4.464 ± 0.257	14.8	1.800
1375	60	2.529 ± 0.054	14.3	4.298 ± 0.081	19.2	1.769
1375	80	3.120 ± 0.167	17.2	4.235 ± 0.210	19.2	1.116
1375	100	2.944 ± 0.235	17.1	—	—	—
1475	0	1.836 ± 0.026	7.1	1.936 ± 0.058	18.8	0.100
1475	20	1.895 ± 0.076	14.5	1.837 ± 0.023	8.0	-0.058
1475	40	2.015 ± 0.110	11.4	1.989 ± 0.109	10.7	-0.026
1475	50	2.145 ± 0.138	10.8	2.159 ± 0.009	29.5	0.014
1475	60	2.397 ± 0.140	13.9	2.279 ± 0.116	14.4	-0.118
1475	80	2.663 ± 0.124	14.9	3.052 ± 0.261	17.6	0.389
1475	100	2.971 ± 0.039	25.5	4.053 ± 0.356	23.7	1.082

Note: All tabulated simulation values use n = 3 independent trajectories.

Additional Spatial Composition Profiles. For the three PBEsol composition-mismatched calculations at 50 at.% Cu overall, spatial Cu-concentration profiles were averaged over the final 20% of the recorded NPH profile trajectory. Using the fixed bulk windows employed for the spatial-profile analysis, the late-time solid/liquid compositions are 19.4/80.1, 30.5/70.9, and 40.6/60.0 at.% Cu for the nominal 20/80, 30/70, and 40/60 cases, respectively. Thus, the imposed phase compositions remain largely preserved in the bulk, while composition changes are localized primarily to the interfacial regions. These fixed-window, final-20%-averaged values differ slightly from the terminal, structurally assigned central-half phase compositions reported in Table S6 because the spatial and temporal

analysis windows differ. Table S6 should be used for terminal composition changes and mismatch-retention percentages; Figure S10 illustrates the late-time spatial profiles.

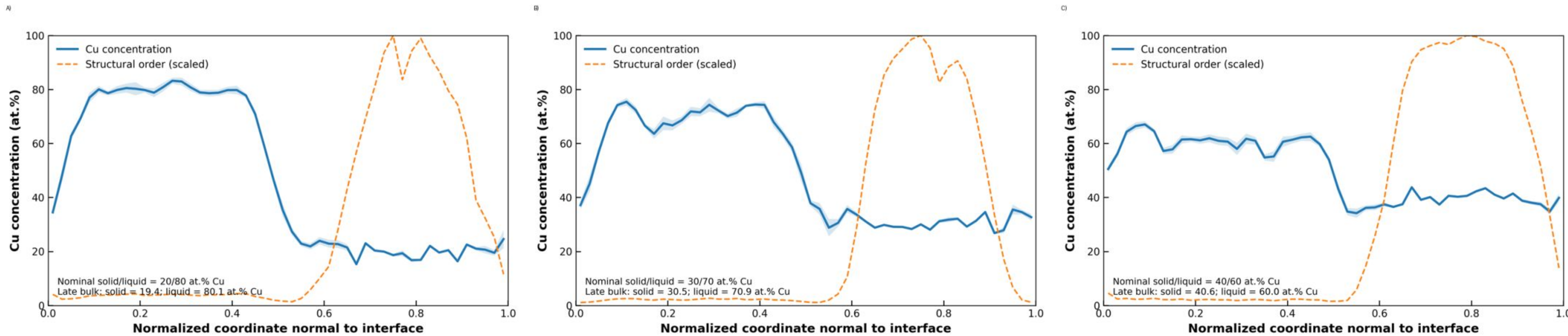

Figure S10. Spatially resolved Cu concentration profiles across the solid–liquid interface for partitioned PBEsol two-phase coexistence simulations at 50 at.% Cu overall. (A) 20/80, (B) 30/70, and (C) 40/60 nominal solid/liquid Cu compositions. Profiles were averaged over the final 20% of the NPH trajectory. The dashed curve shows the scaled structural-order profile to identify the solid and liquid regions. The imposed bulk compositions are retained closely, and compositional changes occur primarily across the diffuse interfacial region.

Finite-Temperature Solid Mixing and Harmonic Vibrational Thermodynamics. The additional ordered-solid calculations provide finite-temperature energetic benchmarks that complement the 0 K cohesive-energy analysis in Figure 3 of the main text. The mixing enthalpies are positive at all three intermediate compositions and both temperatures (Figure S11 and Table S12). The largest values occur at 50 at.% Cu, where PBE predicts 135.6 ± 0.8 and 134.0 ± 0.7 meV atom^{-1} at 700 and 800 K, respectively, compared with 134.1 ± 0.9 and 133.8 ± 0.8 meV atom^{-1} for PBEsol. The close agreement at equiatomic composition shows that the energetic penalty associated with the ordered crystal persists at finite temperature and depends only weakly on the exchange–correlation functional over this temperature interval. These results characterize the specified ordered structures and do not establish their equilibrium stability relative to compositionally partitioned terminal solids.

Table S12. Finite-temperature mixing enthalpies of ordered crystalline Cu–Ag configurations at 700 and 800 K. All values are referenced to the pure crystalline Ag and Cu endpoints calculated using the same functional and temperature. Uncertainties are propagated as block-based standard errors. The pure-element mixing enthalpies are zero by definition. We visually classified all final configurations as ordered crystalline structures.

Functional	Cu (at.%)	700 K	800 K
PBE	25	60.05 ± 1.35	62.28 ± 0.83
PBE	50	135.59 ± 0.81	134.04 ± 0.69
PBE	75	87.03 ± 0.68	86.72 ± 0.72
PBEsol	25	70.15 ± 0.99	69.51 ± 0.83
PBEsol	50	134.13 ± 0.92	133.78 ± 0.75
PBEsol	75	88.75 ± 0.77	87.90 ± 0.54

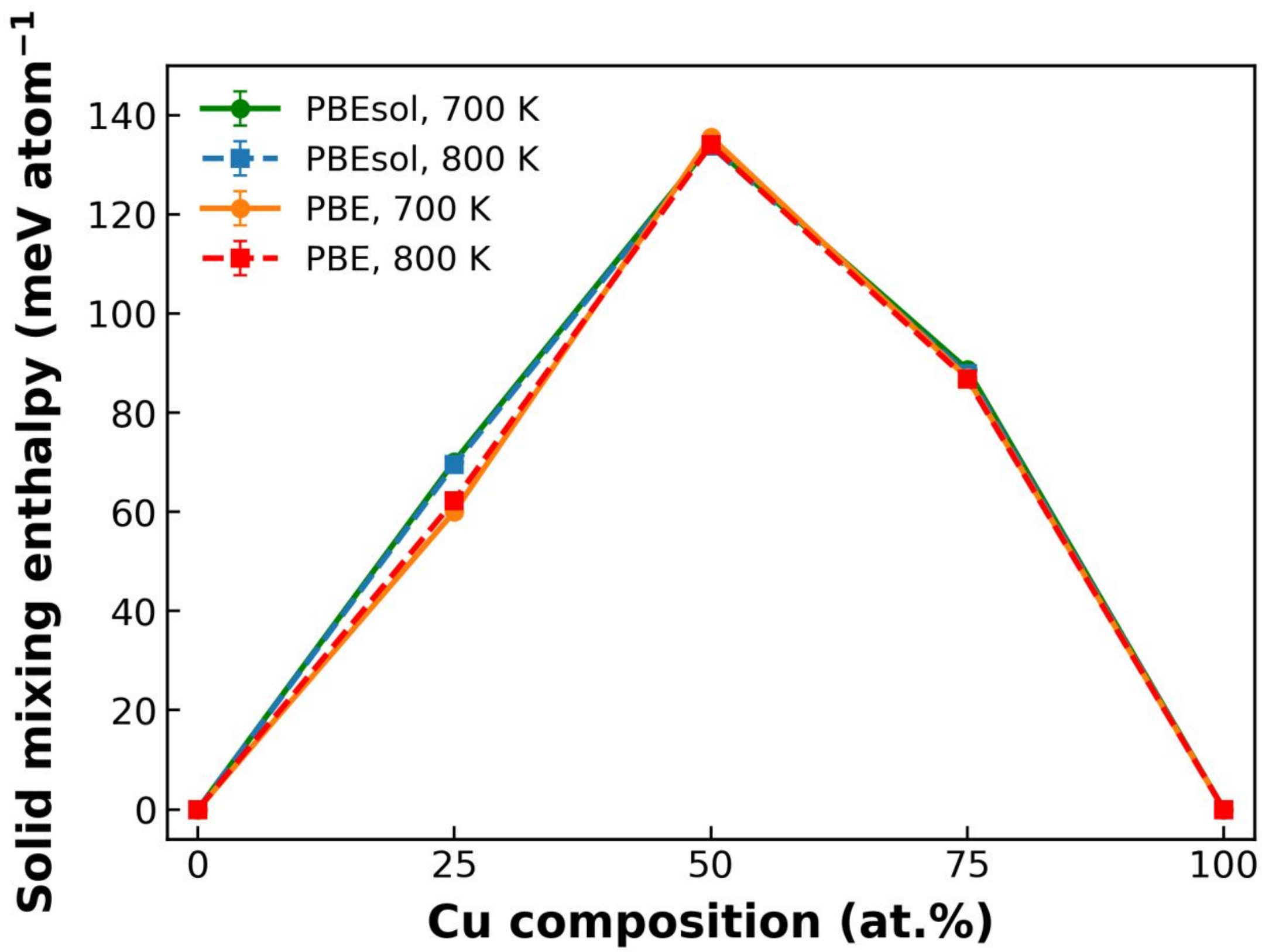

Figure S11. Composition-dependent mixing enthalpy of ordered crystalline Cu–Ag calculated using the PBE- and PBEsol-trained DNPs at 700 and 800 K. Enthalpies are referenced to the corresponding pure crystalline endpoints at each temperature and functional. Error bars indicate propagated block-based standard errors. All intermediate-composition mixing enthalpies are positive, with the largest values at 50 at.% Cu.

Harmonic phonon calculations provide a complementary measure of the vibrational contribution to crystalline mixing. To align the harmonic analysis with the finite-temperature ordered-solid calculations, we report results at 700 and 800 K, where the corresponding ordered configurations remain crystalline in the molecular-dynamics simulations. Three distinct quantities are reported. The classical MD mixing enthalpy, ΔHmixMD(T), is given in Table S12. The zero-point mixing energy, ΔEZPE,mix, is absent from classical MD and may be added separately to define an approximate ZPE-corrected classical mixing enthalpy, ΔHmixMD+ZPE(T) = ΔHmixMD(T) + ΔEZPE,mix. In contrast, ΔFvib,mix(T) is the harmonic vibrational contribution to the mixing free energy and contains ΔEZPE,mix together with the finite-temperature harmonic contribution, including −TΔSvib,mix; ΔFvib,mix does not contain ΔHmixMD. At 50 at.% Cu, ΔEZPE,mix is −3.11 meV atom^{-1} for PBE and −3.66 meV atom^{-1} for PBEsol. The corresponding ZPE-corrected MD mixing enthalpies are 132.48 and 130.93 meV atom^{-1} for PBE and 130.47 and

130.12 meV atom⁻¹ for PBEsol at 700 and 800 K, respectively, while ΔFvib,mix is −23.25/−26.53 meV atom⁻¹ for PBE and −27.10/−30.93 meV atom⁻¹ for PBEsol. The comparatively small ZPE correction therefore does not alter the conclusion that ordered crystalline mixing remains strongly endothermic, whereas the finite-temperature harmonic vibrational contribution favors crystalline mixing. The crystalline vibrational mixing entropies are positive (3.16–3.17 J mol⁻¹ K⁻¹ (per mole of atoms) for PBE and 3.68–3.69 J mol⁻¹ K⁻¹ (per mole of atoms) for PBEsol). At 50 at.% Cu, these entropies correspond to −TΔSvib,mix = −22.9/−26.3 meV atom⁻¹ for PBE and −26.7/−30.6 meV atom⁻¹ for PBEsol at 700/800 K, respectively, explicitly demonstrating a substantial favorable vibrational-entropy contribution to crystalline mixing at this composition. Neither ΔHmixMD+ZPE nor ΔFvib,mix is a complete solid–liquid mixing free energy.

Table S13. Relationship among the classical MD mixing enthalpy, the zero-point-corrected classical mixing enthalpy, and the harmonic vibrational mixing free energy for ordered crystalline Cu–Ag. ΔH_{mix}^{MD} is the finite-temperature classical MD mixing enthalpy. $\Delta H_{mix}^{MD+ZPE} = \Delta H_{mix}^{MD} + \Delta E_{ZPE,mix}$ adds only the quantum zero-point mixing correction. $\Delta F_{vib,mix}$ is a separate harmonic vibrational free-energy contribution that contains $\Delta E_{ZPE,mix}$ and the finite-temperature vibrational term, including $-T\Delta S_{vib,mix}$, but does not contain ΔH_{mix}^{MD}. All values are in meV atom⁻¹. $\Delta F_{vib,mix}$ should not be added directly to either ΔH_{mix}^{MD} or ΔH_{mix}^{MD+ZPE}; none of these quantities represents a complete solid–liquid free-energy difference.

Functional	T (K)	Cu (at.%)	$\Delta E_{ZPE,mix}$ (meV/atom)	ΔH_{mix}^{MD} (meV/atom)	ΔH_{mix}^{MD+ZPE} (meV/atom)	$\Delta F_{vib,mix}$ (meV/atom)
PBE	700	25	−0.832	60.05 ± 1.35	59.22	−5.097
PBE	700	50	−3.105	135.59 ± 0.81	132.48	−23.249
PBE	700	75	−2.131	87.03 ± 0.68	84.90	−13.612
PBE	800	25	−0.832	62.28 ± 0.83	61.45	−5.810
PBE	800	50	−3.105	134.04 ± 0.69	130.93	−26.528
PBE	800	75	−2.131	86.72 ± 0.72	84.59	−15.524
PBEsol	700	25	−1.188	70.15 ± 0.99	68.96	−7.286
PBEsol	700	50	−3.660	134.13 ± 0.92	130.47	−27.104
PBEsol	700	75	−1.582	88.75 ± 0.77	87.17	−9.407
PBEsol	800	25	−1.188	69.51 ± 0.83	68.32	−8.308
PBEsol	800	50	−3.660	133.78 ± 0.75	130.12	−30.925
PBEsol	800	75	−1.582	87.90 ± 0.54	86.32	−10.724

Table S14. PBEsol phonon supercell-size convergence for crystalline vibrational mixing. Values compare 4 × 4 × 4 and 6 × 6 × 6 finite-displacement supercells at 0, 700, and 800 K. The maximum absolute difference in $\Delta F_{vib,mix}$ is 0.0019 meV atom^{-1}, demonstrating effective convergence of the 4 × 4 × 4 results used for the PBE/PBEsol comparison in Table S13.

Cu (at.%)	T (K)	4×4×4 (meV/atom)	6×6×6 (meV/atom)	6×6×6 − 4×4×4 (meV/atom)	ΔS_{mix} difference (J mol^{-1} K^{-1}; per mole of atoms)
25	0	−1.187652	−1.187625	+0.000027	0.000000
25	700	−7.286347	−7.285935	+0.000412	−0.000055
25	800	−8.307550	−8.307090	+0.000460	−0.000055
50	0	−3.659565	−3.659590	−0.000025	0.000000
50	700	−27.104095	−27.104680	−0.000585	+0.000080
50	800	−30.925480	−30.926150	−0.000670	+0.000080
75	0	−1.582437	−1.582465	−0.000027	0.000000
75	700	−9.406772	−9.408375	−0.001603	+0.000221
75	800	−10.724450	−10.726280	−0.001830	+0.000221

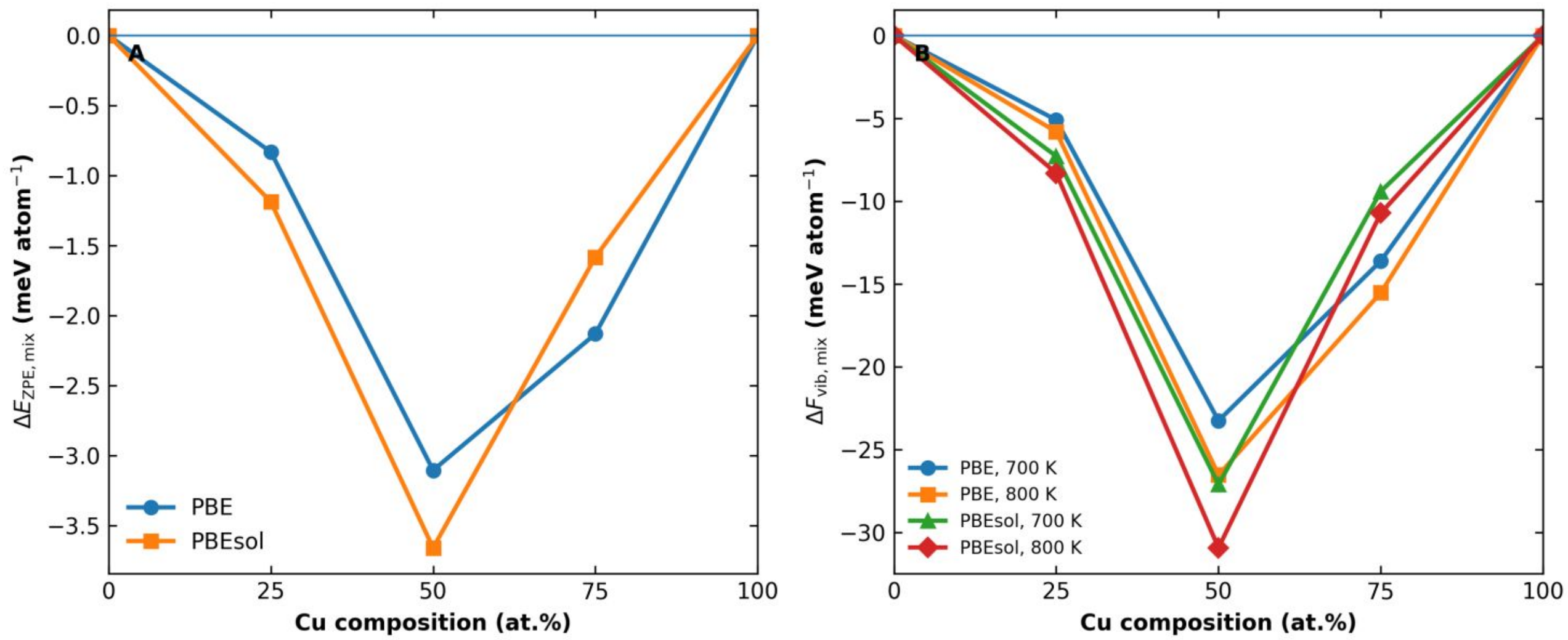

Figure S12. Harmonic vibrational contributions to crystalline Cu–Ag mixing. (A) Zero-point vibrational mixing energy, $\Delta E_{ZPE,mix}$. (B) Harmonic vibrational mixing free energy, $\Delta F_{vib,mix}$, at 700 and 800 K for PBE- and PBEsol-trained DNPs using 4 × 4 × 4 phonon supercells. $\Delta F_{vib,mix}$ contains the zero-point and finite-temperature harmonic vibrational terms but not the classical MD mixing enthalpy. The corresponding $\Delta H_{mix}{}^{MD}$ and $\Delta H_{mix}{}^{MD+ZPE}$ values are reported in Table S13. Negative vibrational values indicate stabilization of the mixed crystalline configurations relative to the pure crystalline endpoints; PBEsol supercell convergence is documented in Table S14.

REFERENCES

(1) Kresse, G.; Furthmuller, J. Efficient iterative schemes for ab initio total-energy calculations using a plane-wave basis set. *Phys. Rev. B - Condens. Matter Mater. Phys.* **1996**, *54* (16), 11169–11186.
(2) Kresse, G.; Joubert, D. From ultrasoft pseudopotentials to the projector augmented-wave method. *Phys. Rev. B - Condens. Matter Mater. Phys.* **1999**, *59* (3), 1758–1775.
(3) Blochl, P. E. Projector augmented-wave method. *Phys. Rev. B - Condens. Matter Mater. Phys.* **1994**, *50* (24), 17953–17979.
(4) Perdew, J. P.; Burke, K.; Ernzerhof, M. Generalized Gradient Approximation Made Simple. *Phys. Rev. Lett.* **1996**, *77* (18), 3865–3868.
(5) Perdew, J. P.; Ruzsinszky, A.; Csonka, G. I.; Vydrov, O. A.; Scuseria, G. E.; Constantin, L. A.; Zhou, X.; Burke, K. Restoring the Density-Gradient Expansion for Exchange in Solids and Surfaces. *Phys. Rev. Lett.* **2008**, *100* (13), 136406.

(6) Wang, H.; Zhang, L.; Han, J.; Weinan, E. DeePMD-kit: A deep learning package for many-body potential energy representation and molecular dynamics. *Comput. Phys. Commun.* **2018**, *228*, 178–184.
(7) Andolina, C. M.; Saidi, W. A. Predicting melting temperatures across the periodic table with machine learning atomistic potentials. *Digit. Discov.* **2024**, *3* (7), 1421–1429.
(8) Murnaghan, F. D. Finite deformations of an elastic solid. *Am. J. Math.* **1937**, *59* (2), 235–260.
(9) Birch, F. Finite elastic strain of cubic crystals. *Phys. Rev.* **1947**, *71* (11), 809.
(10) Wang, H.; Zhang, Y.; Zhang, L.; Wang, H. Crystal structure prediction of binary alloys via deep potential. *Front. Chem.* **2020**, *8*, 589795.
(11) Plimpton, S. Fast Parallel Algorithms for Short-Range Molecular Dynamics. *J. Comput. Phys.* **1995**, *117* (1), 1–19.
(12) Nitol, M. S.; Iriarte, M. J. E.; Dickel, D. E.; Fensin, S. J. Evaluating moment tensor potential in Ag--Cu alloy: Accuracy, transferability, and phase diagram fidelity. *Phys. Rev. Mater.* **2026**, *10* (2), 023602.
(13) Wisesa, P.; Andolina, C. M.; Saidi, W. A. Assessing Melting Points from Machine Learning Interatomic Potentials Using PBE and PBEsol Exchange-Correlation Functionals. *J. Chem. Phys.* **2026**, *165* (4).
(14) Rosenbrock, C. W.; Gubaev, K.; Shapeev, A. V.; Pártay, L. B.; Bernstein, N.; Csányi, G.; Hart, G. L. Machine-learned interatomic potentials for alloys and alloy phase diagrams. *npj Comput. Mater.* **2021**, *7* (1), 24.
(15) Byggmästar, J.; Nordlund, K.; Djurabekova, F. Simple machine-learned interatomic potentials for complex alloys. *Phys. Rev. Mater.* **2022**, *6* (8), 083801.
(16) Marchand, D.; Curtin, W. Machine learning for metallurgy IV: A neural network potential for Al-Cu-Mg and Al-Cu-Mg-Zn. *Phys. Rev. Mater.* **2022**, *6* (5), 053803.
